# Boosting urea electrooxidation on oxyanion-engineered nickel sites via inhibited water oxidation

**DOI:** 10.1038/s41467-023-41588-w

**Published:** 2023-09-20

**Authors:** Xintong Gao, Xiaowan Bai, Pengtang Wang, Yan Jiao, Kenneth Davey, Yao Zheng, Shi-Zhang Qiao

**Affiliations:** School of Chemical Engineering, The University of Adelaide, Adelaide, SA Australia

**Keywords:** Energy, Electrocatalysis

## Abstract

Renewable energy-based electrocatalytic oxidation of organic nucleophiles (e.g.methanol, urea, and amine) are more thermodynamically favourable and, economically attractive to replace conventional pure water electrooxidation in electrolyser to produce hydrogen. However, it is challenging due to the competitive oxygen evolution reaction under a high current density (e.g., >300 mA cm^−2^), which reduces the anode electrocatalyst’s activity and stability. Herein, taking lower energy cost urea electrooxidation reaction as the model reaction, we developed oxyanion-engineered Nickel catalysts to inhibit competing oxygen evolution reaction during urea oxidation reaction, achieving an ultrahigh 323.4 mA cm^−2^ current density at 1.65 V with 99.3 ± 0.4% selectivity of N-products. In situ spectra studies reveal that such in situ generated oxyanions not only inhibit OH^−^ adsorption and guarantee high coverage of urea reactant on active sites to avoid oxygen evolution reaction, but also accelerate urea’s C − N bond cleavage to form CNO ^−^ intermediates for facilitating urea oxidation reaction. Accordingly, a comprehensive mechanism for competitive adsorption behaviour between OH^−^ and urea to boost urea electrooxidation and dynamic change of Ni active sites during urea oxidation reaction was proposed. This work presents a feasible route for high-efficiency urea electrooxidation reaction and even various electrooxidation reactions in practical applications.

## Abstract (web summary)

Urea electrooxidation reaction is of great importance in energy related applications and devices but is limited by competing oxygen evolution reaction. Here, the authors report oxyanion-engineered nickel catalysts that can achieve efficient urea oxidation while suppressing oxygen evolution reaction.

## Introduction

Abundant urea in wastewater and human urine is a practically promising alternative energy carrier and (indirect) hydrogen (H_2_) storage chemical^1–4^. The six-electron transferred electrocatalytic urea oxidation reaction (UOR) is an important anode half-reaction in energy-related applications including, direct urea /urine fuel cells (DUFC)^5–7^ and urea-assistant water electrolysers^8–10^. The thermodynamic potential for UOR coupled with cathode hydrogen evolution reaction is theoretically 0.37 V (vs. reversible hydrogen electrode, RHE), a value significantly less than that of 1.23 V for water electrolysis^8,10^. Therefore, UOR has a significant potential to reduce voltage input for H_2_ production by replacing traditional oxygen evolution reaction (OER) in the anode of a water electrolyser. Recently, nickel (Ni)-based electrocatalysts have been reported for UOR^11–15^. In most cases, these catalysts undergo surface reconstruction into nickel oxyhydroxide (NiOOH) species during UOR because of the anodic potential and electrolyte’s hydroxyl anions, which serve as the active species for UOR^11,14,16^. However, the derived NiOOH are also highly active species for OER, an undesired side reaction toward UOR^17,18^. More importantly, OER will over-oxidize NiOOH to high-valent nickel^19,20^, thereby degrading the electrode stability and reducing further UOR efficiency. This competition between UOR and OER is more significant in practical UOR-related applications, especially with high operating potential and large current density devices^3,14,21^.

The competition between UOR and OER originates from the adsorption of OH^−^. UOR in an alkaline environment needs OH^–^ to proceed through a series of proton-coupled electron transfers and to cleavage the C–N bonds in urea molecule^21,22^, however, excessive OH^–^ adsorption on the active sites will block adsorption of urea reactant and accelerate competing OER. Although this kind of competition has been mentioned in some anodic reactions including, methanol electrooxidation and 5-hydroxymethylfurfural electrooxidation^23,24^, it has not been explored in UOR. Importantly, there is no efficient strategy for catalysts to overcome this competition. Practically, given UOR and OER is six-electron and four-electron transferred reaction, respectively^3,16,25^, competitive OER will, theoretically, reduce anode current density by >30%. Therefore, the design for catalysts to obviate OER in the UOR at a high potential to achieve a large apparent current density and excellent electrode stability is essential for the development of the next generation of urea-assisted industrial water electrolysers.

Here, we realized highly selective and active UOR on oxyanions (sulfur, phosphorus, and selenium) coordinated nickel catalyst. Electrochemical kinetics measurements, combined with in situ spectroscopy characterizations and potential dependent density function theory (DFT) calculations indicate that the critical roles of such in situ generated oxyanions: (1) it protects Ni active sites with high coverage of urea reactant via inhibiting adsorption of OH^−^ anion to obviate OER; (2) it promotes the C − N bond cleavage of urea molecules to generate more CNO ^−^ intermediates for boosting UOR. As a result, the optimized sulfur oxyanion nickel (Ni-SO_X_) sample exhibits an ultrahigh current density of 323.4 mA cm^–2^ and a 99.3 ± 0.4% UOR selectivity at 1.65 V (vs. RHE). This versatile and feasible oxyanion-engineered strategy solves competitive adsorption of organic reactant and hydroxyl anion on the active sites and opens a fresh avenue for the design of high-performance electrocatalysts under large current density operations, and therefore, is expected to be extended to other organic electrooxidation reactions proceeding in aqueous electrolyte to obviate competing OER.

## Results

### Catalyst preparation and characterization

**Fig. 1:**
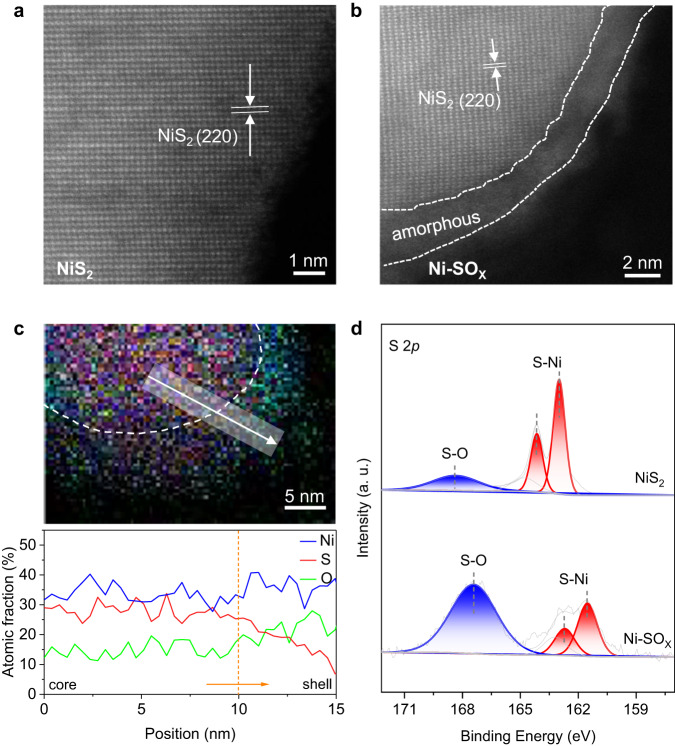
Activation of NiS_2_ to Ni-SO_X_ under anodic potential. HRTEM image of **a** NiS_2_ and **b** Ni-SO_X_ catalyst. **c** EDX elemental mapping (upper) and linear scan (lower) for Ni-SO_X_. **d** S 2*p* XPS curves for NiS_2_ and Ni-SO_X_ catalyst.

Pre-catalyst NiS_2_ was prepared via a sulfuration of Ni(OH)_2_ precursors (Supplementary Fig. 1, and Methods section). High-angle annular dark-field scanning transmission electron microscopy (HAADF-STEM) and X-ray diffraction (XRD) confirmed a pure phase of NiS_2_ (PDF: 03-065-3325) with nanoparticles morphology. Energy-dispersive X-ray (EDX) mapping images evidenced that Ni and S atoms are uniformly distributed (Fig. 1a and Supplementary Fig. 2). After an electrochemical activation at a potential of 1.45 V for 600 s in urea-containing alkaline solution (1 M KOH + 0.33 M urea, Supplementary Fig. 3), derived electrocatalyst was obtained, which exhibited a core-shell structure with a crystalline NiS_2_ core and a 2–5 nm thick amorphous shell (Fig. 1b). EDX mapping images and line analyses showed that the Ni and O elements present mainly in the surface layer with small amount of S, evidencing that the sulfur species exist with nickel (oxy)hydroxide in the shell (Fig. 1c and Supplementary Fig. 4)^26^. The derived electrocatalyst was denoted therefore Ni-SO_X_. Compared with fresh NiS_2_, Ni-SO_X_ exhibited weaker peaks for Ni-S binding and a stronger S-O peak in the X-ray photoelectron spectroscopy (XPS) patterns (Fig. 1d), demonstrating that partial S in the matrix was oxidized to sulfate interacting with nickel (oxy)hydroxide in the shell of Ni-SO_X_^26,27^. This finding is also confirmed by the Near-edge X-ray absorption fine structure spectroscopy (NEXAFS) for S K-edge, in which Ni-SO_X_ exhibited a weaker peak for S_2_^2−^ and a stronger peak for SO_4_^2−^ compared with NiS_2_ (Supplementary Fig. 5)^28,29^. For comparison, NiO_X_ was also synthesized *via* electrooxidation of pristine Ni(OH)_2_ catalysts without sulfuration, which presented typical nickel (oxy)hydroxide on the surface shell (Supplementary Figs. 6 and 7).

### Electrocatalytic UOR performance

**Fig. 2:**
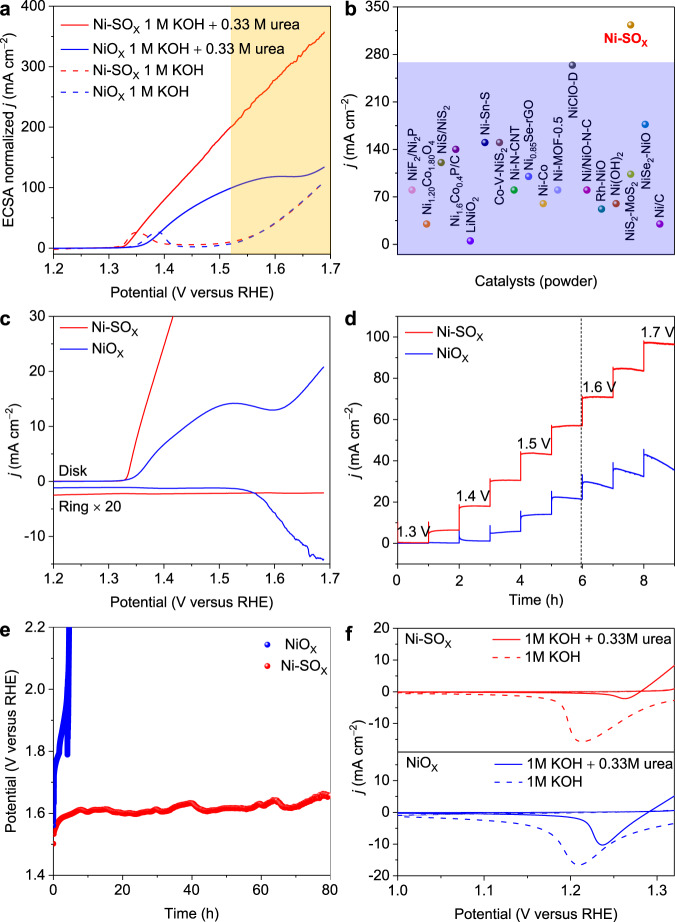
Electrocatalytic UOR performance for Ni-SO_X_ and NiO_X_. **a** ECSA-normalized LSV curves in 1 M KOH solution with (solid line), or without (dash line) 0.33 M urea. **b** Comparison of current density on Ni-SO_X_ with reported powder catalysts under a 1.50 − 1.65 V potential zone for UOR. **c** In situ evaluation of O_2_ on Ni-SO_X_ and NiO_X_ using RRDE in 1 M KOH with 0.33 M urea. **d** Multi-step chronopotentiometric test at different potentials from 1.30 to 1.70 V. **e** Long-term chronopotentiometric stability determined at current density 50 mA cm ^−2^. **f** Comparison of Ni^3+^ reduction peaks for Ni-SO_X_ and NiO_X_ in 1 M KOH with (solid line) or without (dash line) 0.33 M urea.

UOR performance for Ni-SO_X_ and NiO_X_ powder electrodes were tested in 1 M KOH with 0.33 M urea solution *via* a typical three-electrode system. Both the Ag/AgCl (saturated KCl) with a salt bridge and Hg/HgO were used as the reference electrodes. As is shown in the electrochemically active surface area (ECSA) normalized linear sweep voltammetry (LSV) curve (Fig. 2a and Supplementary Figs. 8, 9), both Ni-SO_X_ and NiO_X_ exhibit higher anodic current toward UOR (solid line) than that for OER (dash line). Overall OER performance for Ni-SO_X_ and NiO_X_ is similar, however UOR performance for the former is significantly better than that for the latter (confirmed by a smaller Tafel curves in Supplementary Fig. 10). At 1.65 V, UOR current density on Ni-SO_X_ is as high as 323.4 mA cm^−2^, which is *ca*. 3 times greater than that on NiO_X_. Significantly, this finding is amongst ‘best’ reported performance for powder Ni-based UOR electrocatalysts (Fig. 2b and Supplementary Table 1). To exclude the effect of different nanostructures on the final UOR performance, we also synthesized Ni(OH)_2_* nanoparticles precursors with similar morphology to NiS_2_ nanoparticles precursors (Supplementary Fig. 11). After the electrochemical activation, the ECSA-normalized UOR performance of Ni-SO_X_ remained superior to NiO_X_* (Supplementary Figs. 12, 13). This finding confirms that the significant UOR current density for Ni-SO_X_ is because of the critical roles of sulfur oxyanion dopant and, importantly, not the nanostructure of the catalyst itself.

It is noteworthy that the UOR current density for NiO_X_ exhibits current passivation at a high potential (>1.50 V, yellow-colour zone) (Fig. 2a), while that for Ni-SO_X_ exhibits a potential-current linear relationship. To exclude the effect of diffusion limit, we performed the polarization tests at different rotating speeds of 400, 800, and 1600 rpm in 1 M KOH with 0.33 M urea electrolyte (Supplementary Fig. 14). The LSV curves for Ni-SO_X_ and NiO_X_ exhibited almost constant current densities with an increasing rotating speed. This finding evidences that UOR behaviors for Ni-SO_X_ and NiO_X_ are diffusion-independent. Because this current passivation in NiO_X_ is exhibited near the OER zone, we hypothesized that it is current consumption by the 4e ^−^ transferred OER rather than the 6e ^−^ transferred UOR. This was confirmed via a rotating ring-disk electrode (RRDE) where UOR /OER occurred on the disk and generated O_2_ (if any) was reduced on the Pt ring electrode. As is shown in Fig. 2c, compared with the significant reduction current for NiO_X_ under potential >1.50 V, no ring current is exhibited with Ni-SO_X_ in the test range. This difference demonstrates that UOR selectivity on Ni-SO_X_ was *ca*. 100%, whereas a certain amount of OER exhibits on NiO_X_ under a high potential.

As expected, the partial OER influences the stability of the whole electrocatalyst. As is shown in Fig. 2d, the current density for NiO_X_ decays significantly from 1.50 V, and the decay rate exceeds 20% at 1.70 V, in contrast to Ni-SO_X_ which remains stable at all potentials. This difference is evidenced also in the long-term stability of Ni-SO_X_ and NiO_X_ via chronopotentiometry at 50 mA cm ^−2^ (Fig. 2e), in which NiO_X_ remained stable for only 3 h, significantly less than the 80 h for Ni-SO_X_. The performance degradation for NiO_X_ is likely because of the generation of high valance Ni species (> +3) associated with OER (Fig. 2f). Specifically, in 1 M KOH medium, Ni-SO_X_ and NiO_X_ have similar high state Ni^3+^ reduction peaks because of continuous oxidation potentials and OER. However, in 1 M KOH with 0.33 M urea, Ni-SO_X_ exhibited a much smaller Ni^3+^ reduction peak than NiO_X_, evidencing that the Ni species maintain a relatively low valance state on Ni-SO_X_ because of UOR (we will discuss the reason in later sections), which leads to it exhibiting greater stability than NiO_X_.

### UOR/OER selectivity quantification

**Fig. 3:**
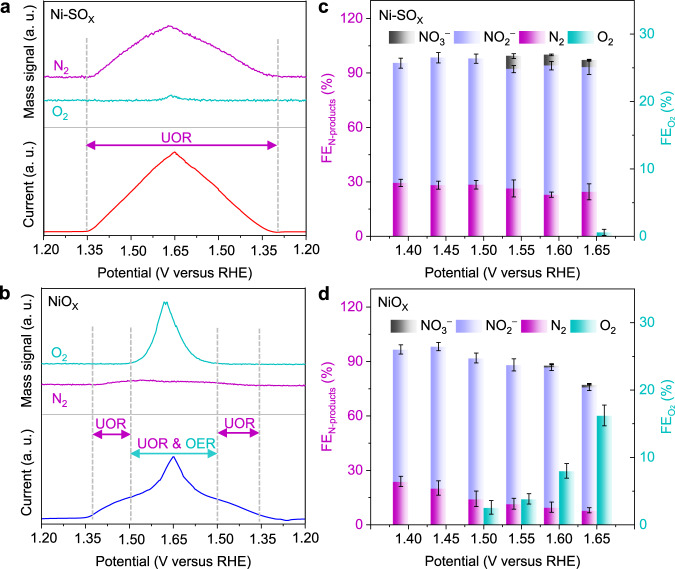
UOR /OER selectivity quantification. In situ detection of the representative gaseous products of UOR (N_2_) and OER (O_2_) via DEMS (upper) and the corresponding current acquired in 1 M KOH with 0.33 M urea (lower, without *iR* correction) on **a** Ni-SO_X_ and **b** NiO_X_. FEs for different UOR /OER products under different potentials on **c** Ni-SO_X_ and **d** NiO_X_. Error bars correspond to the standard deviation of three independent measurements.

In situ differential electrochemical mass spectrometry (DEMS) was used to determine the potential dependent UOR/OER selectivity on the electrocatalysts (Fig. 3a, b). For Ni-SO_X_, from 1.31 to 1.65 V, the N_2_ signal was detected without attenuation, with O_2_ negligible. In comparison, the N_2_ signal weakened and O_2_ signal gradually increased for NiO_X_ from 1.50 V. This finding is in good agreement with experimental results of RRDE. The potential dependent Faradic efficiency (FEs) for each ion and gaseous product from UOR and OER (if any) were quantified via ion chromatography (IC) and gas chromatography (GC) (Fig. 3c, d and Supplementary Fig. 15). Error bars indicate the standard deviation based on three independent measurements. The N-containing products from UOR are nitrite (NO_2_^−^), N_2_, nitrate (NO_3_^−^), and cyanate (CNO ^−^ ), of which NO_2_^−^ is main. The generated N_2_ may follow the equation: CO(NH_2_)_2_ + OH^−^ → CO_2_ + N_2_ + H_2_O + 6e^−^, and the typical reaction pathway: urea adsorption; dehydrogenation of N − H; C − N bond breakage; N − N coupling; N_2_ and CO_2_ desorption^10,12,16,21^. The C-containing products from UOR are CNO ^−^ and carbonate (CO_3_^2 −^ ). The FEs for UOR products are computed based on N-containing products (N_2_, NO_2_^−^, and NO_3_^−^). For Ni-SO_X_, the FEs for N-containing products (FE_N-products_) from UOR remained above 95 ± 4% at all potentials with few OER products (O_2_). In contrast, for NiO_X_, the FE_N-products_ monotonically decreased, and ${{{\rm{FE}}}}_{{{{\rm{O}}}}_{2}}$ increased with increasing applied potential. Specifically, UOR selectivity was reduced to 82.6 ± 0.7% at 1.65 V for NiO_X_, whilst that for Ni-SO_X_ remained up to 99.3 ± 0.4%. This difference in OER /UOR selectivity for NiO_X_ and Ni-SO_X_ leads to the disparity in the apparent current (Fig. 2a). Significantly, this UOR selectivity on Ni-SO_X_ is one of the highest reported Ni-based electrocatalysts under large current density operation conditions (Supplementary Table 2).

### Dynamic state change of Ni during UOR

**Fig. 4:**
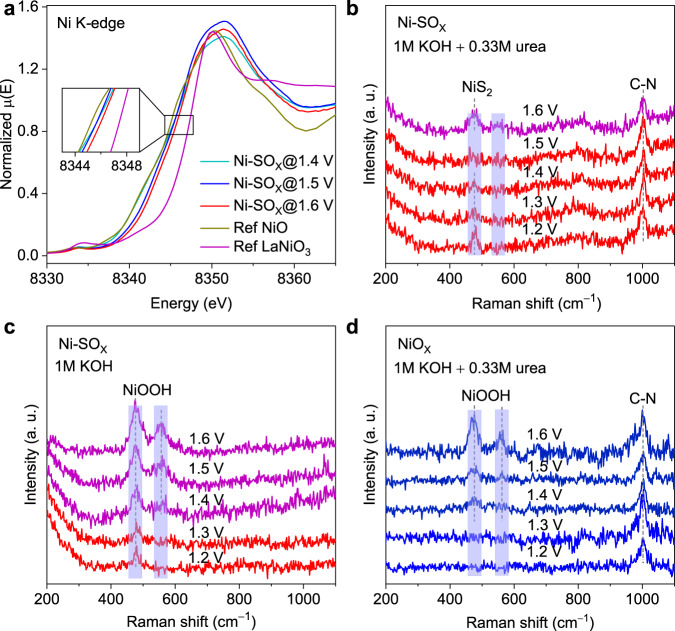
Dynamic state change of Ni during UOR. **a** In situ Ni K-edge XANES spectra for Ni-SO_X_ under varying potential during UOR. Inset, magnified absorption edge region. In situ Raman spectra for Ni-SO_X_ electrode in 1 M KOH **b** with, and **c** without, 0.33 M urea at applied potentials. **d** In situ Raman spectra for NiO_X_ electrode in 1 M KOH with 0.33 M urea.

To determine the origin for the high UOR activity, selectivity, and stability on Ni-SO_X_, we performed in situ X-ray absorption near-edge structure (XANES) and in situ Raman spectra to gain some insights into the influence of oxyanion incorporation on active Ni sites during UOR. With applied potential increasing, the adsorption edge of Ni-SO_X_ is positively shifted towards higher energy than that of Ref NiO, but still lower than that of Ref LaNiO_3_, which is evidence that the state of Ni in Ni-SO_X_ increased from +2 and remained below +3 during UOR up to 1.60 V (Fig. 4a). And a linear relationship between the Ni K-edge absorption edge and the Ni oxidation states in Ni-SO_X_@1.4 V, Ni-SO_X_@1.5 V, Ni-SO_X_@1.6 V, Ref NiO, and Ref LaNiO_3_ is plotted (Supplementary Fig. 16)^30,31^. The in situ Raman spectra for Ni-SO_X_ show that, with 0.33 M urea in the electrolyte, there is not a high state of NiOOH accumulation (note that the peak at 475 cm ^− 1^ is attributed to *A*_g_ mode of NiS_2_ in the core of Ni-SO_X_^32,33^ and remained almost unchanged at different voltage) (Fig. 4b). NiOOH species were detected in pure 1 M KOH electrolyte in which OER is dominated (Fig. 4c), which is in good agreement with the results from the CV curves (Fig. 2f). This can be explained that when there is urea in the electrolyte, the potential induced NiOOH rapidly catalyzes the nucleophile urea molecule to oxidation products and spontaneously reduces to Ni^2+^ ^21,22,34^. Conversely, accumulation of abundant NiOOH is found on NiO_X_ in 1 M KOH with 0.33 M urea electrolyte (Fig. 4d). This evidences that NiO_X_ has ‘slow’ UOR kinetics, causing accumulation of NiOOH, which then acts as active species for OER, leading to O_2_ evolution.

### UOR mechanism analyses

**Fig. 5:**
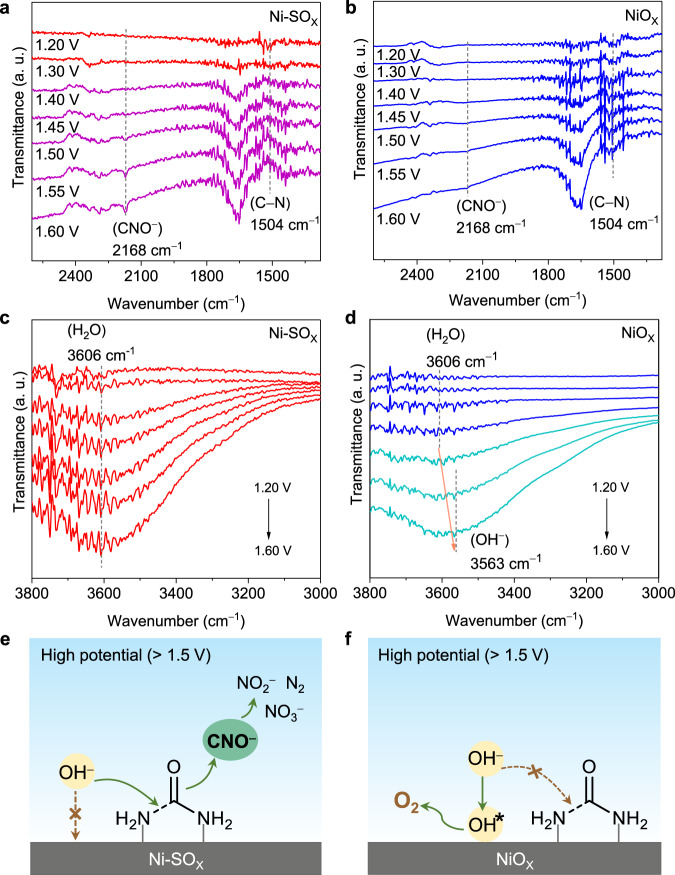
UOR mechanism analyses. **a**–**d** In situ ATR-IR spectra in potential window 1.20 to 1.60 V for Ni-SO_X_ and for NiO_X_ in different ranges. **e**, **f** Schematic for competing adsorption of urea and hydroxyl on Ni-SO_X_ and NiO_X_ surfaces.

In situ ATR-IR spectroscopy was used to explore how sulfur oxyanion dopant promotes UOR kinetics and inhibits OER on Ni-SO_X_. As is shown in Fig. 5a, b, a urea’s C − N stretching vibration peak at ∼1504 cm^−1^ was found in both Ni-SO_X_ and NiO_X_ in the initial stage^35,36^.

With the increase in potentials, this band on Ni-SO_X_ decreased more than that on NiO_X_, evidencing that the C − N bond can be more readily cleaved on Ni-SO_X_. This is evidenced also by a stronger CNO ^−^ intermediate vibration peak at ∼ 2168 cm^−1^ on Ni-SO_X_^37,38^. Then, we constructed relatively matched NiOOH-SO_4_ and NiOOH models to simulate active sites of Ni-SO_X_ and NiO_X_ during UOR and speculated possible pathways of urea oxidation to main product NO_2_^−^ based on the observed intermediate (Supplementary Fig. 17). The computational results show that C − N bond cleavage is promoted on NiOOH-SO_4_ compared with NiOOH, which is consistent with in situ ATR-IR data (Supplementary Fig. 18). To further explore the origin of this C-N bond cleavage promotion on Ni-SO_X_, the surface adsorption behaviour of the two catalysts was investigated. In the initial stage, an identical stretching mode for the O–H group in H_2_O molecule was found for both Ni-SO_X_ and NiO_X_ (Fig. 5c, d)^39,40^. As the potential increased to 1.50 V, these peaks exhibited a red-shift from 3606 to 3563 cm ^− 1^ on NiO_X_, whilst there was no change for Ni-SO_X_. This red-shift, together with potentials confirms generation of OH* as a surface-adsorbed species, according to the Stark tuning phenomenon^40,41^. Importantly, given that the adsorption of OH* on the active sites of catalysts is the first step in OER, this atomic level observation explains the selectivity of OER on NiO_X_ at a high potential. However, the surface of Ni-SO_X_ is covered by urea molecules which then readily are attacked by OH ^−^ species from the alkaline electrolyte, thereby promoting the C-N cleavage of urea for boosted UOR and obviating adsorption of OH* for suppressed OER (Fig. 5e, f). An experimental kinetics study exhibits that the reaction order of UOR for NiOOH-SO_4_ with respect to OH ^−^ concentration is 0.65, a value significantly less than that for NiOOH of 1.87, further confirming that UOR for NiOOH-SO_4_ exhibits a weaker dependence on OH ^−^ concentration (Supplementary Fig. 19)^8,42^.

To validate the inhibiting role of oxyanion toward OH ^−^ adsorption on more Ni-based electrocatalysts, we further tested the UOR selectivity of other Ni compounds including NiSe_2_ and Ni_5_P_4_ (Supplementary Fig. 20), which was derived to Ni-SeOx and Ni-POx during UOR. HAADF-STEM images and XPS spectra evidence that selenium and phosphorus oxyanion doped amorphous nickel (oxy)hydroxides are also formed on the surface of Ni-SeO_4_ and Ni-PO_4_ (Supplementary Figs. 21–23). As expected, selenium and phosphorus oxyanion doping boosts UOR activity and inhibits OER as sulfur (Supplementary Fig. 24).

### UOR /OER Competition Mechanism

**Fig. 6:**
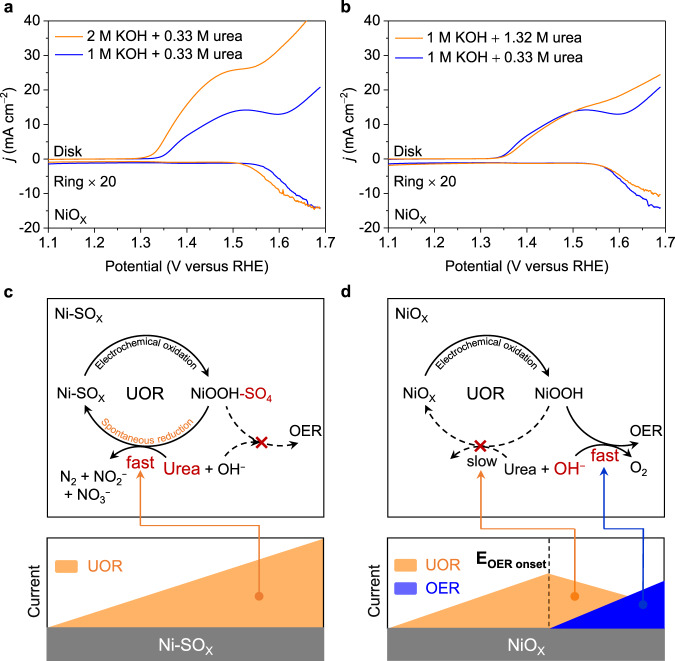
UOR /OER competition mechanism. **a** RRDE curves for NiO_X_ obtained in different concentration of KOH with 0.33 M urea. **b** RRDE curves for NiO_X_ obtained in 1 M KOH with different concentrations of urea. **c** Representation for UOR mechanism on Ni-SO_X_ accompanying adsorption of reactants and dynamic evolution of active sites under high potential (>1.50 V). **d** Representation of UOR and OER mechanism on NiO_X_ accompanying adsorption of reactants and dynamic evolution of active sites under high potential (>1.50 V).

To manipulate the competing adsorption of urea and OH ^−^ , electrochemical tests were performed in electrolytes containing different concentrations of reactants. As is shown in Fig. 6a, a greater OH ^−^ concentration in electrolyte leads to more significant OER competition for NiOx, whilst having no apparent effect for Ni-SO_X_ (Supplementary Fig. 25). The increase in urea concentration slightly attenuates but does not eliminate competition between UOR and OER on NiO_X_ (Fig. 6b), evidencing that the surface of NiO_X_ is mainly covered by OH ^−^ species but not urea reactant, leading to dominant OER selectivity.

Based on above electrochemical tests and in situ spectroscopic studies, a possible UOR mechanism on two kinds of electrocatalysts including adsorption of reactants and dynamic evolution of active sites is proposed. When the applied potential is low (<1.50 V), Ni-SO_X_ and NiO_X_ are derived to, respectively, NiOOH-SO_4_ and NiOOH on the surface through electrochemical oxidation (Supplementary Fig. 26). The generated NiOOH-SO_4_ and NiOOH catalyze the nucleophile urea molecules into N products and spontaneously reduced to low-valent Ni-SO_X_ and NiO_X_, maintaining a good balance of derivatization and reduction. Compared with NiOOH, NiOOH-SO_4_ makes the C − N of urea more readily cleaved, resulting in boosted UOR. Once the potential is applied over 1.50 V (Fig. 6c, d), derived NiOOH is attacked by OH ^−^ to generate high valance Ni species (≥ 3). These over-oxidized Ni species significantly participate in competing OER, which makes less NiOOH oxidizing urea, and spontaneous reduction to NiO_X_ is slowed, leading to UOR-OER competition. However, derived NiOOH-SO_4_ can inhibit the adsorption of OH ^−^ , making it covered by sufficient nucleophile urea molecules, thereby obviating the generation of higher-valent OER-active species. Simultaneously, the repulsion of OH ^−^ on NiOOH-SO_4_ promotes cleavage of C − N of urea molecules and generates more CNO ^−^ intermediates, resulting in faster UOR kinetics and more facile spontaneous reduction into Ni-SO_X_ as a clean active site for the next derivatization and reduction cycle.

## Discussion

In summary, we achieved ultrahigh activity, selectivity, and stability of UOR *via* constructing oxyanion-engineered nickel catalysts (Ni-SO_X_, Ni-PO_X_, and Ni-SeO_X_). Notably, the optimal Ni-SO_X_ exhibited ultrahigh 323.4 mA cm ^− 2^ UOR current density at 1.65 V with nearly 100% selectivity of N-products. The combination of diverse in situ spectroscopic measurements and DFT calculations evidenced the essential roles of oxyanion for UOR. On one hand, it inhibits the competitive adsorption of OH ^−^ on the Ni active sites to avoid OER. On the other hand, it accelerates urea’s C − N bond cleavage to form CNO ^−^ intermediates for boosting UOR. Correspondingly, we proposed a comprehensive mechanism for competitive adsorption behaviour between OH ^−^ and urea to boost UOR and dynamic change of Ni active sites. We expect that this strategy will aid future research in practical urea electrolysis, and other multi-electron organic molecule oxidation coupled with cathodic hydrogen evolution for overall atomic economy and additional green energy production.

## Methods

### Synthesis of Ni(OH)_2_, NiS_2_, Ni_5_P_4_, and NiSe_2_

In typical synthesis for Ni(OH)_2_ catalyst, 5 mmol nickel (II) nitrate hexahydrate (Ni(NO_3_)_2_·6H_2_O) and 10 mmol hexamethylenetetramine (HMT) were dissolved in 35 mL of deionized (DI) water under vigorous stirring for 30 min to form a transparent solution. The mixture was transferred to a 50 mL Teflon-lined autoclave, sealed, and heated at 120 °C for 12 h. The resulting powder was washed with ethanol /water and dried overnight to obtain Ni(OH)_2_ powder. NiS_2_ catalyst was obtained from Ni(OH)_2_ via a simple calcination. In detail, 20 mg of Ni(OH)_2_ and 0.8 g sulfur (S) powder were placed in two separate porcelain boats and put into a tube-furnace, with S powder on the upstream. After flushing with Ar for ~30 min, the sample was heated to 350 °C at a rate 2 °C min^−1^ in Ar for 2 h and cooled naturally to room temperature (RT). The Ni_5_P_4_ catalyst was obtained via replacing 0.8 g S with 0.8 g hypophosphite monohydrate (NaH_2_PO_2_•H_2_O). The NiSe_2_ catalyst was obtained via replacing 0.8 g S with 0.4 g Selenium (Se) powder heated to 450 °C at a rate 20 °C min^−1^ in Ar for 1 h.

### Synthesis of NiO_X_, Ni-SO_X_, Ni-PO_X_ and Ni-SeO_x_

NiO_X_, Ni-SO_X_, Ni-PO_X_ and Ni-SeO_4_ catalysts were prepared via electrochemical activation of Ni(OH)_2_, NiS_2_, Ni_5_P_4_ and NiSe_2_ at a potential of 1.45 V for 600 s in 1 M KOH with 0.33 M urea aqueous solution.

### Characterizations

X-Ray Powder Diffraction (XRD) data were collected on a Rigaku MiniFlex 600 X-Ray Diffractometer. HAADF-STEM images were obtained using an FEI Titan G2 80–300 microscope at 300 kV equipped with a probe corrector. EDX imaging was carried out with an FEI Titan Themis 80–200 microscope equipped with a SuperX detector. X-ray photoelectron spectroscopy (XPS) analyses were conducted under ultra-high vacuum on a Kratos Axis Ultra with a Delay Line Detector photoelectron spectrometer using an aluminum monochromatic X-ray source. XPS data were corrected using the C1*s* line at 284.8 eV. In situ XAS data were collected on XAS beamline of Australian Synchrotron (ANSTO, Melbourne) using a home-made cell and processed via the Athena program. In situ Raman spectroscopy data were obtained using a Via-Reflex spectrometer (Renishaw) with a laser excitation wavelength of 532 nm. The measured potential was in the range of 1.20 to 1.60 V *vs*. RHE controlled by a CHI 760E electrochemical workstation.

### In situ DEMS measurement

A 60 nm gold-sputtered PTFE membrane was used as working electrode substrate. Electrocatalyst ink was added onto the gold-sputtered membrane and dried at RT to a uniform layer. Real-time generated gaseous products (N_2_ and O_2_) during the Cyclic voltammetry (CV) test were pumped to in situ DEMS system (HPR-40, Hiden Analytical Ltd.), and the signal with mass-to-charge ratio of 28, 32, was obtained. CV test was performed at a rate 10 mV s ^−1^ under 1 M KOH with 0.33 M urea.

### In situ Attenuated Total Reflectance Infrared (ATR-IR) spectroscopy measurement

In situ ATR-IR measurements were performed on a Thermo-Fisher Nicolet iS20 equipped with a liquid nitrogen-cooled HgCdTe (MCT) detector and a VeeMax III ATR accessory (Pike Technologies). A silicon prism coated with Au film (60°, PIKE Technologies) was mounted in a PIKE electrochemical three-electrode cell with an Ag/AgCl reference electrode (Pine Research) and a Pt counter electrode. Catalysts were sprayed onto the Au film as a working electrode. During testing, the electrolyte (1 M KOH with 0.33 M urea solution) was purged with Ar continuously. A CHI 760E electrochemical workstation was used to perform chronoamperometric tests from 1.20 to 1.60 V vs. RHE. ATR-IR curves were concurrently collected with 64 scans and a spectral resolution of 4 cm^−1^.

### Electrochemical measurement

Electrochemical data were recorded via a CHI 760E electrochemical workstation. Tests were conducted in a gas-tight three-electrode H-cell at 25 °C (with Ar purge). Anode and cathode compartments were separated by a proton exchange membrane (Nafion 117). For the working electrode, 3.5 mg catalyst, 1 mg carbon black, and 10 μL 5 wt% Nafion solution were dispersed in 400 μL ethanol. Catalyst dispersion was dropped on a carbon-fibre paper or a rotating disk electrode as working electrode (1 × 0.5 cm ^−2^, loading mass: ~0.5 mg cm^−2^). The Ag/AgCl (saturated KCl) with a salt bridge and Hg/HgO were used as the reference electrodes, while a graphite rod was used as the counter electrode. The electrolyte was 1 M KOH (or 2 M KOH, 4 M KOH) with and without 0.33 M (or 1.32 M) urea. The linear sweep voltammetry (LSV) and cyclic voltammetry (CV) tests were collected at a scan rate of 5 mV s^−1^ with Ar purging. The estimated electrochemical double layer capacitances (C_dl_) were obtained via CV testing in a non-Faradaic potential region, 1.09 ~ 1.17 V *vs*. RHE at a scan rate of 20, 40, 60, 80, and 100 mV s^−1^. Potentials were converted to reversible hydrogen electrode (RHE) and corrected with 90% *iR*-compensation, except however for stability experiments.

### Product analyses

The gas products (N_2_, O_2_) formed during urea oxidation were analyzed every 15 minutes via on-line gas chromatograph (GC, 8890, Agilent). The GC was fitted with HP-PLOT Q and CP-Molsieve 5 Å PT column (both Agilent), together with thermal conductivity (TCD) and flame ionization (FID) detectors. Liquid nitrate and nitrite (NO_3_^−^ and NO_2_^−^ ) were determined via ion chromatography (IC, Thermo Scientific Dionex Integrion RFIC) equipped with a Dionex IonPac AS19-4 µm 2 × 250 mm column. Calibration-curve standards, containing 5–200 ppm NO_3_^−^/NO_2 _^−^ were prepared from commercially available solution of the anions (1000 ppm, Sigma-Aldrich). FE for the formation of the product was computed from:

1 $${{{{{\rm{FE}}}}}}={{{{{\rm{eF}}}}}} \times {{{{{\rm{n}}}}}}/{{{{{\rm{Q}}}}}}={{{{{\rm{eF}}}}}} \times {{{{{\rm{n}}}}}} / ({{{{{\rm{I}}}}}} \times {{{{{\rm{t}}}}}})$$

in which e is the number of transferred electrons for each product, F Faraday constant, Q charge, I applied current, t reaction time, and n, total product (mole).

Measurements for all products were repeated at least 3 times during each applied potential to exclude any possible errors.

### Reporting summary

Further information on research design is available in the Nature Portfolio Reporting Summary linked to this article.

### Supplementary information

Supplementary Information

Peer Review File

Reporting Summary

### Source data

Source Data

## Data Availability

Data that support findings from this study are available from the corresponding author upon reasonable request. The source data underlying Figs. 1–6 are provided as a Source Data file. Source data are provided in this paper.
