# Supplementary material for: Boosting urea electrooxidation on oxyanion-engineered nickel sites via inhibited water oxidation
Source: Nat Commun. 2023 Sep 20;14:5842. doi: 10.1038/s41467-023-41588-w (PMC10511637; doi:10.1038/s41467-023-41588-w)
Supplement: Supplementary file 1 — Supplementary Information [file 41467_2023_41588_MOESM1_ESM.pdf]

Supplementary Information

Boosting Urea Electrooxidation on Oxyanion-Engineered Nickel Sites *via* Inhibited Water Oxidation

Xintong Gao[1#], Xiaowan Bai[1#], Pengtang Wang[1#], Yan Jiao[1], Kenneth Davey[1], Yao Zheng[1]*, Shi-Zhang Qiao[1]*

[1]School of Chemical Engineering, The University of Adelaide, Adelaide, SA 5005, Australia.

[#]These authors contributed equally to this work.

*Corresponding E-mail: yao.zheng01@adelaide.edu.au; s.qiao@adelaide.edu.au

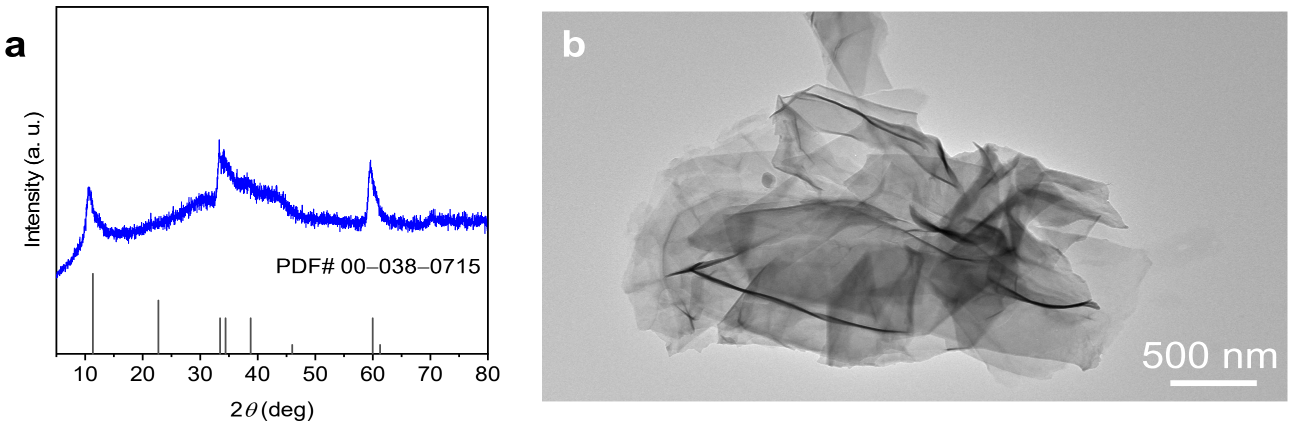

Supplementary Fig. 1 Structural characterization of $Ni(OH)_2$. (a) XRD pattern for and (b) HRTEM image of $Ni(OH)_2$.

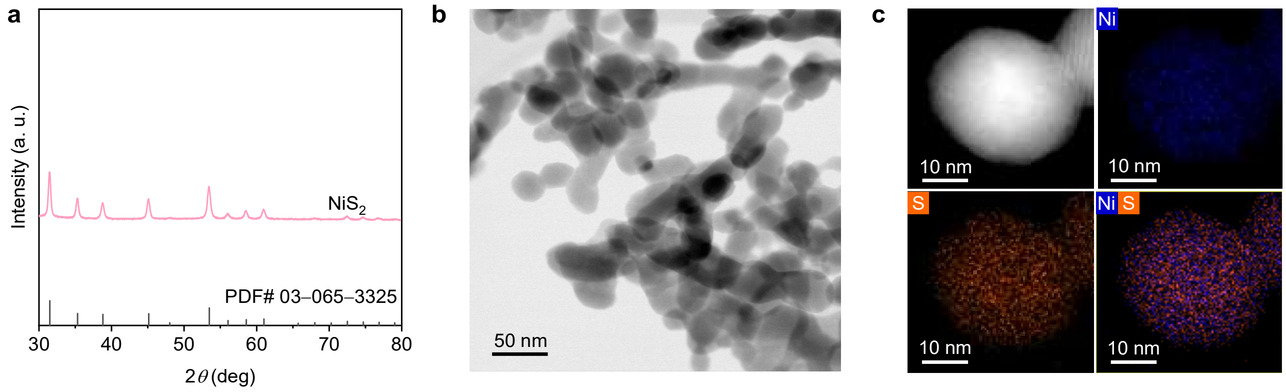

Supplementary Fig. 2 Structural characterization of NiS_2. (a) XRD pattern, (b) HRTEM image and (c) EDX elemental mapping images of NiS_2.

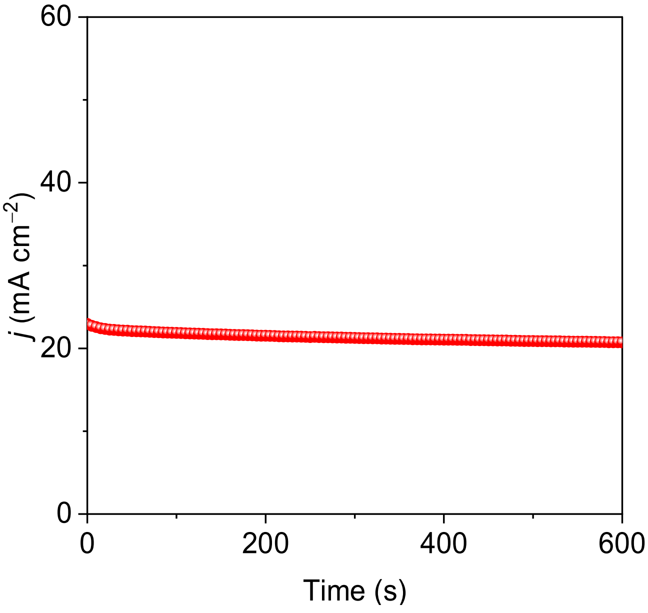

Supplementary Fig. 3 Activation of NiS_2 to Ni-SO_X. I-t curve for converting NiS_2 to Ni-SO_X at 1.45 V for 600 s in 1 M KOH with 0.33 M urea solution.

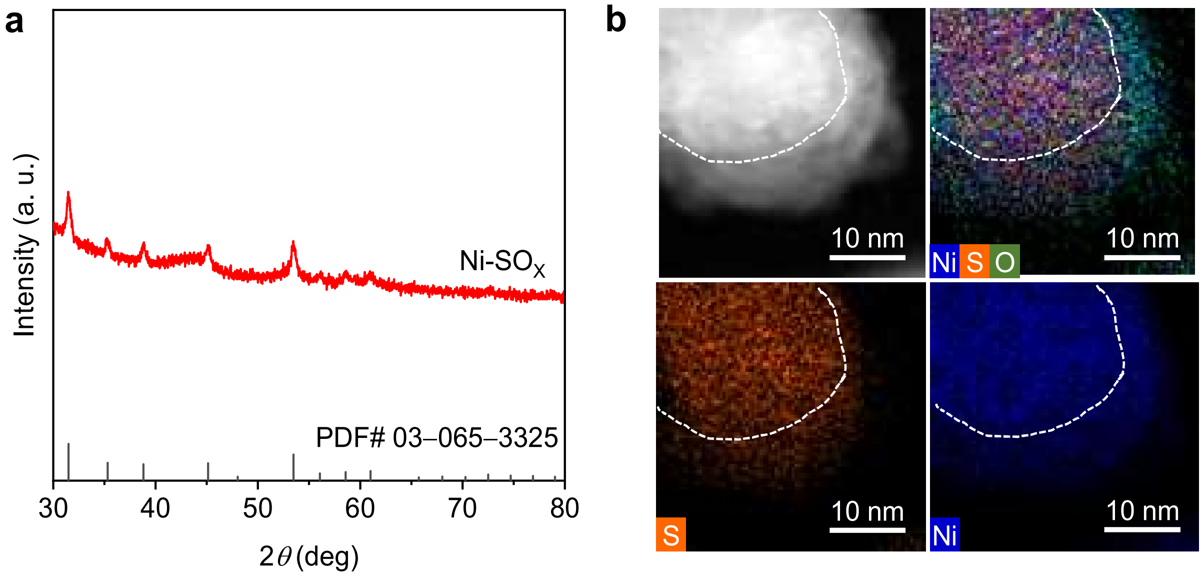

Supplementary Fig. 4 Structural characterization of Ni-SO_X. (a) XRD pattern and (b) EDX elemental mapping for Ni-SO_X.

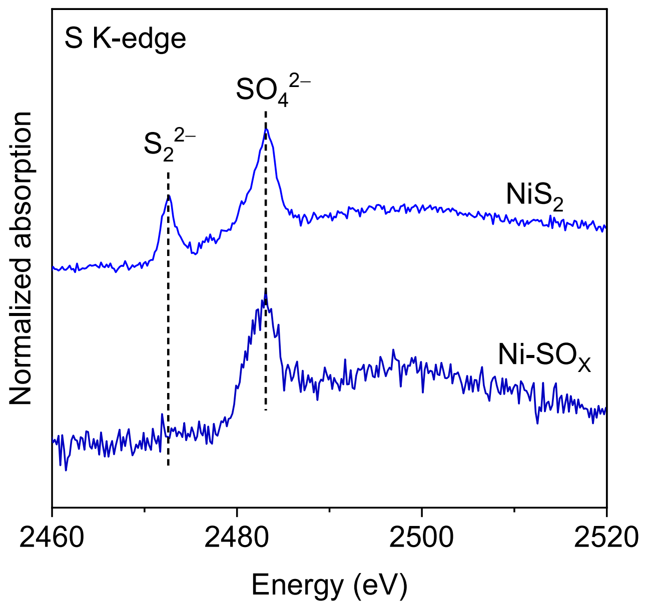

Supplementary Fig. 5 NEXAFS characterization. S K-edge NEXAFS spectra for NiS_2 and Ni-SO_X catalysts.

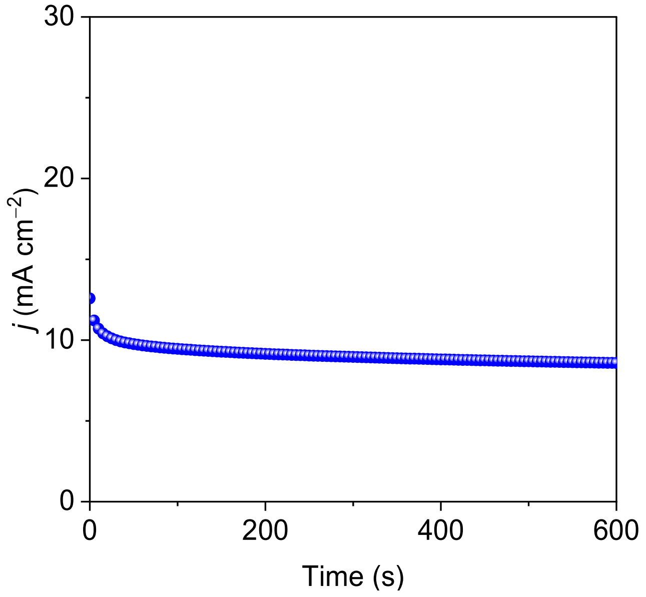

Supplementary Fig. 6 Activation of $Ni(OH)_2$ to NiO_X. I-t curve for converting $Ni(OH)_2$ to NiO_X at 1.45 V for 600 s in 1 M KOH with 0.33 M urea solution.

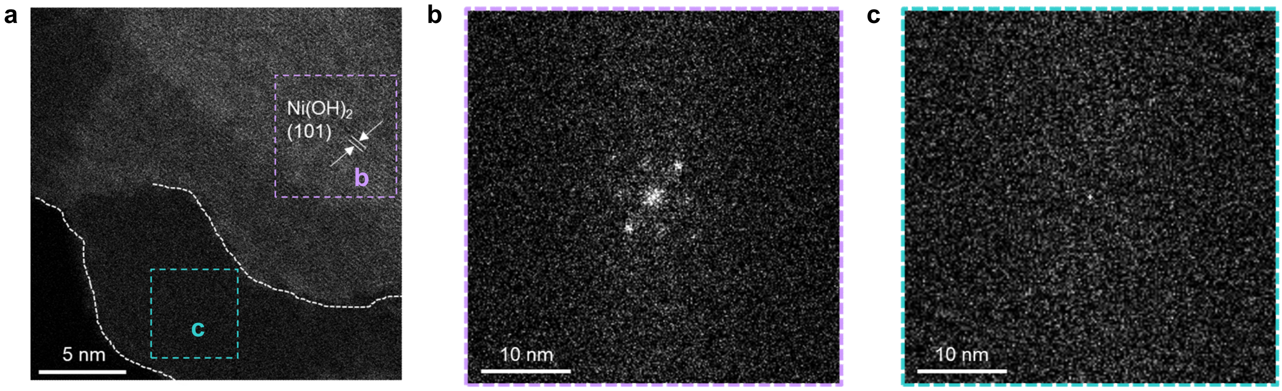

Supplementary Fig. 7 Structural characterization of NiO_X. (a) HRTEM image of NiO_X, (b) Corresponding FFT pattern selected from region b in Supplementary Fig. 7a. (c) Corresponding FFT pattern selected from region c in Supplementary Fig. 7a.

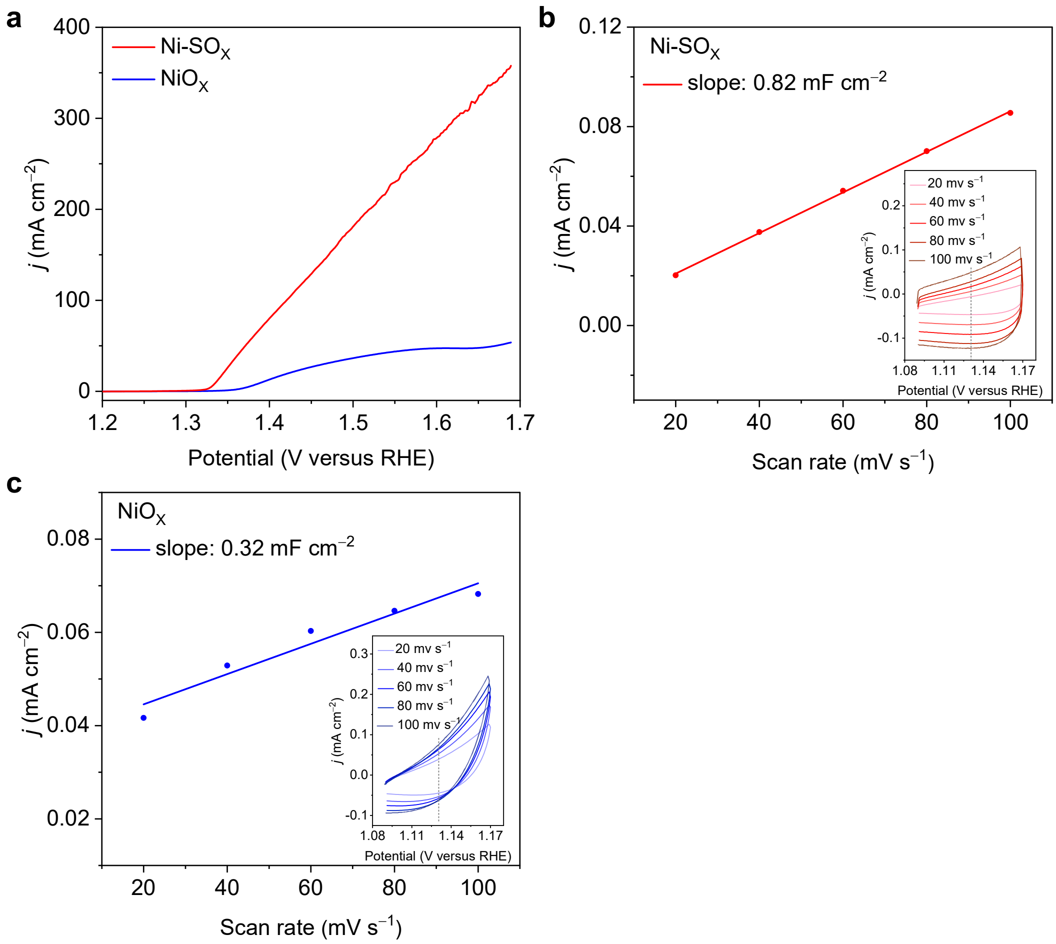

Supplementary Fig. 8 Determination of double-layer capacitance. (a) LSV curve for Ni-SO_X and NiO_X catalyst in 1 M KOH containing 0.33 M urea without ECSA-normalized. (b, c) Electrochemical double-layer capacitance (C_{dl}) for Ni-SO_X and NiO_X, respectively. Inset, CV curve at different scan rates in non-Faradaic capacitance current range.

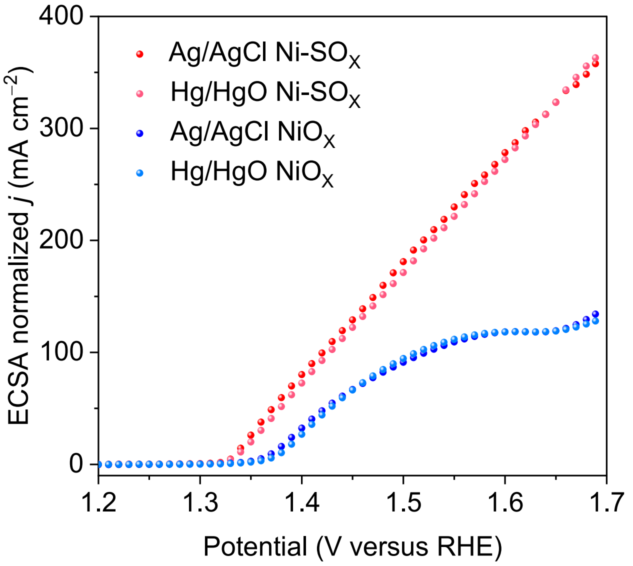

Supplementary Fig. 9 Polarization curves. ECSA-normalized LSV curves in 1 M KOH solution containing 0.33 M urea with Ag/AgCl and Hg/HgO as reference electrodes, respectively.

As is seen in Supplementary Fig. 9, the LSV curves for Ni-SO_X and NiO_X measured with Hg/HgO as the reference electrode are consistent with those of Ag/AgCl (saturated KCl) as the reference electrode. This finding confirms that Ag/AgCl (saturated KCl) electrode is stable in alkaline solution when protected by a salt bridge, that is attributed to the directly contact of Ag/AgCl with the saturated KCl solution rather than the alkaline solution[1,2].

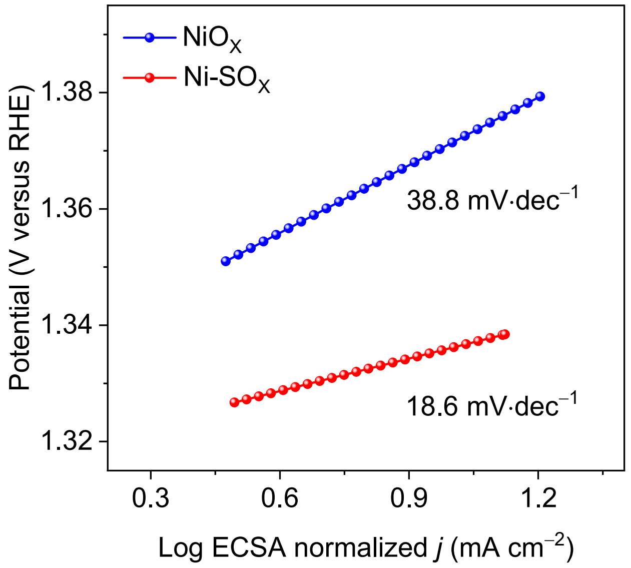

Supplementary Fig. 10 Tafel curve. Tafel slops for Ni-SO_X and NiO_X catalyst during UOR.

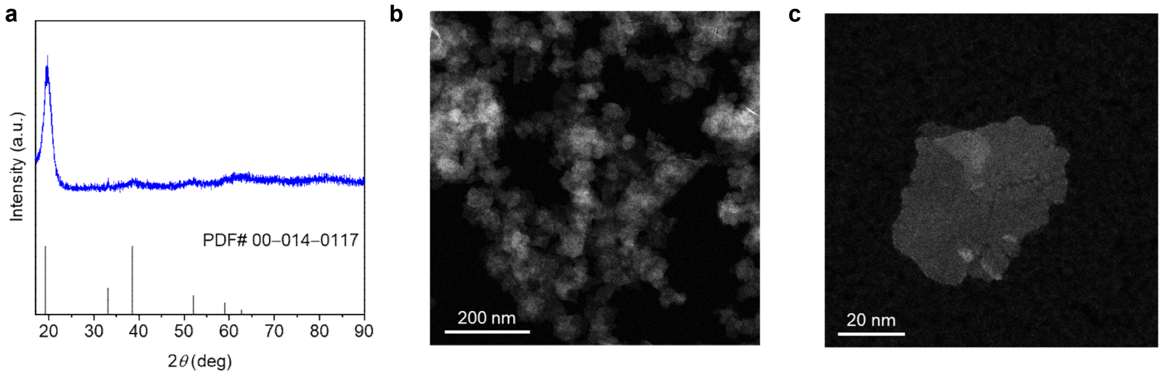

Supplementary Fig. 11 Structural characterization of $Ni(OH)_2$*. (a) XRD pattern, (b) and (c) HRTEM image images of $Ni(OH)_2$*.

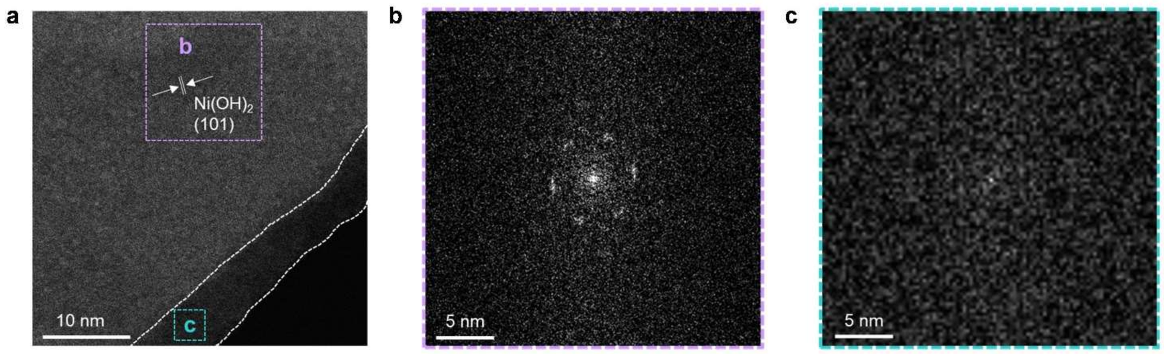

Supplementary Fig. 12 Structural characterization of NiO_X*. (a) HRTEM image of NiO_X*, (b) Corresponding FFT pattern selected from region b in Supplementary Fig. 12a. (c) Corresponding FFT pattern selected from region c in Supplementary Fig. 12a.

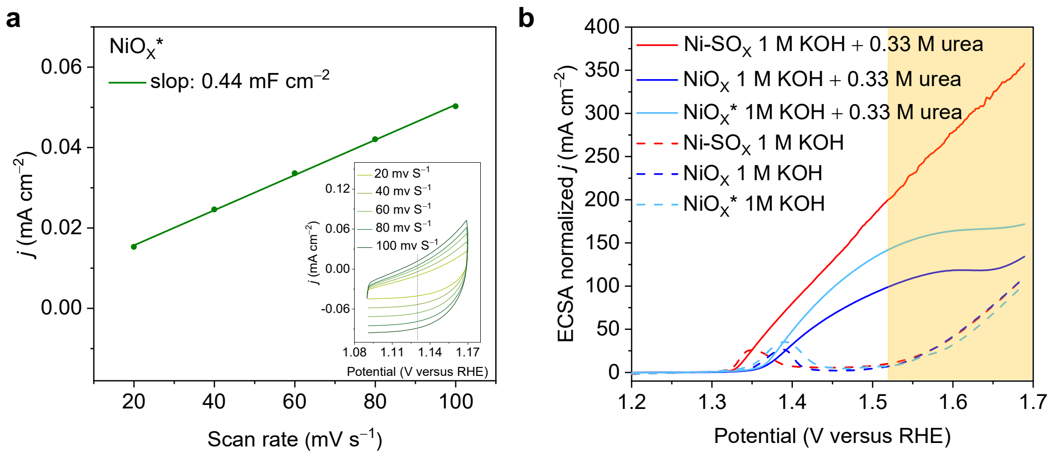

Supplementary Fig. 13 Determination of double-layer capacitance. (a) Electrochemical double-layer capacitance (C_{dl}) for NiO_X*. Inset, CV curve at different scan rates in non-Faradaic capacitance current range. (b) ECSA-normalized LSV curves for Ni-SO_X, NiO_X and NiO_X* in 1 M KOH solution with (solid line), or without (dash line) 0.33 M urea.

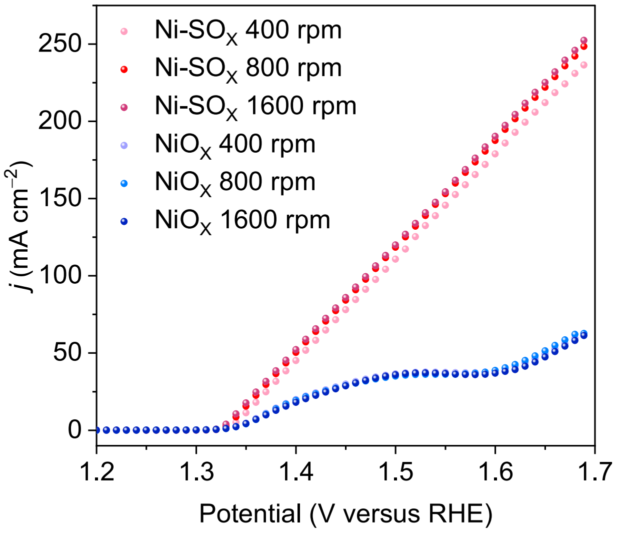

Supplementary Fig. 14 Polarization curves. LSV curves for Ni-SOX and NiOX on a rotating disk electrode (RDE) at different rotation rates (without iR correction).

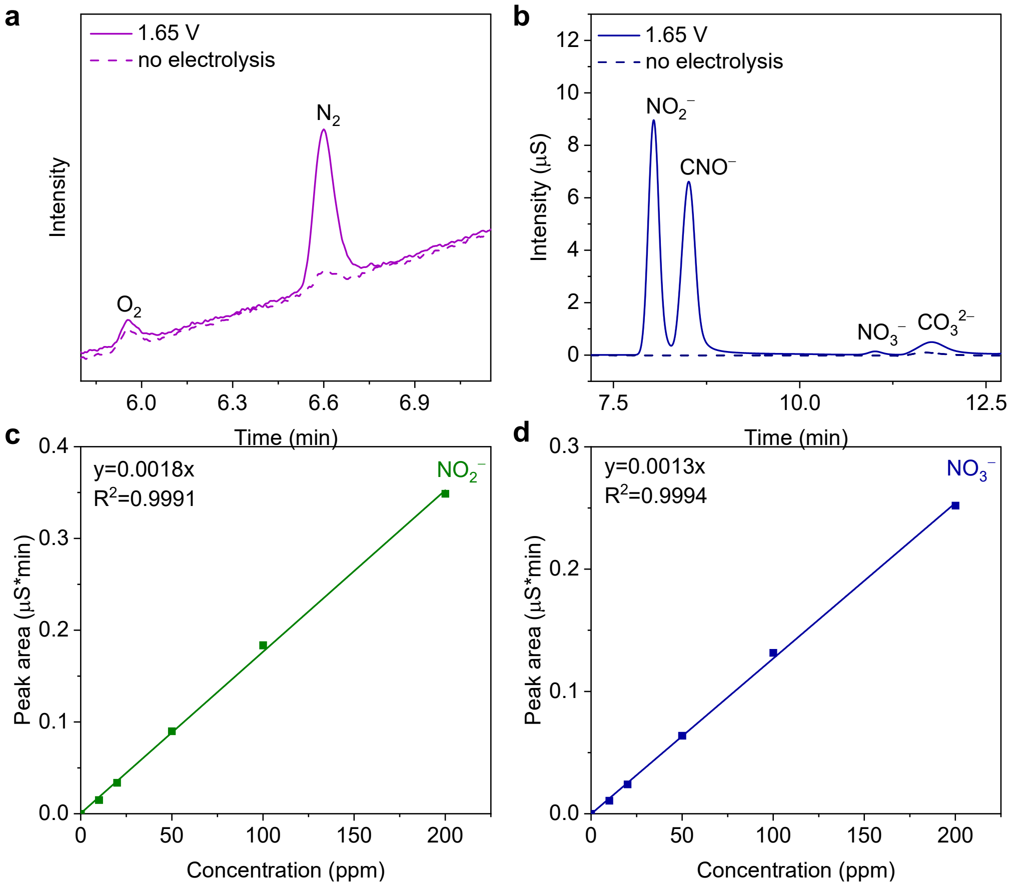

Supplementary Fig. 15 Quantification of products. (a) GC trace for gaseous products during electrochemical UOR 1.65 V on Ni-SOX. (b) IC trace for ion products during electrochemical UOR at 1.65 V on Ni-SOX. Calibration curves for (c) NO_2^- and, (d) NO_3^- *via* IC measurement.

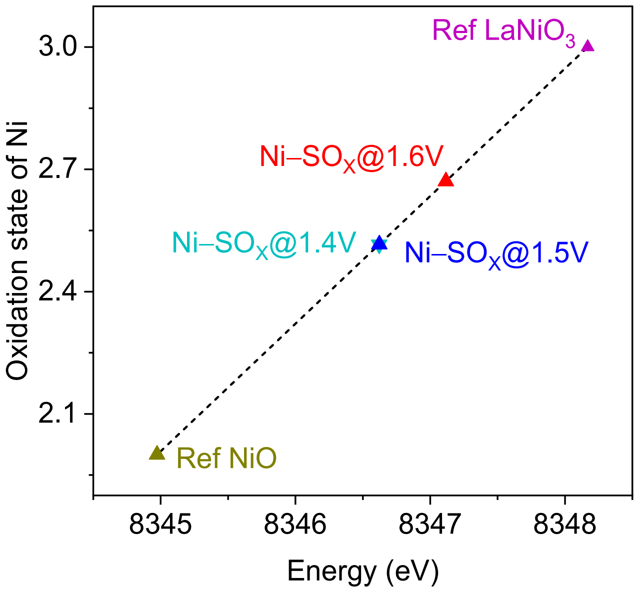

Supplementary Fig. 16 In situ XAS characterization for Ni-SOX catalysts under varying potential during UOR. Relationship between the Ni K-edge absorption edge and the Ni oxidation states in Ni-SOX under varying potential during UOR, reference NiO and $LaNiO_3$.

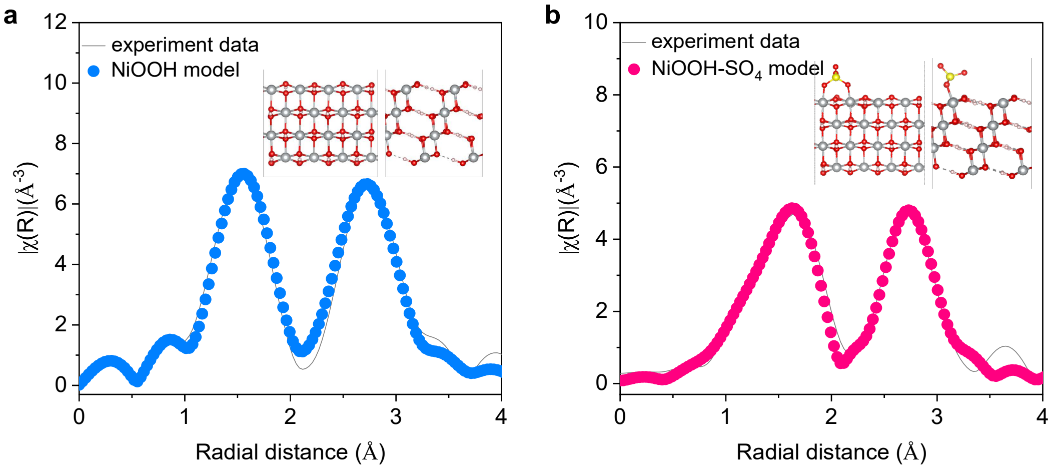

Supplementary Fig. 17 EXAFS fitting for NiOx and Ni-SOx catalysts. (a) EXAFS fitting results of Ni K-edge at k-space of NiOx during UOR under 1.5 V (vs. RHE). The inset shows the relatively matching NiOOH model. (b) EXAFS fitting results of Ni K-edge at k-space of Ni-SOx during UOR under 1.4 V (vs. RHE). The inset shows the relatively matching NiOOH-SO_4 model. The red, gray, light pink, and yellow spheres represent O, Ni, H, and S atoms, respectively.

Although the inability to observe crystal plane of amorphous NiOOH by HRTEM has limited the construction of models, *in situ* extended X-ray absorption fine structure (EXAFS) measurements for catalysts provide a possible solution to assisted-guide the DFT model construction of amorphous NiOOH[3,4]. Therefore, we construct a relatively matching NiOOH model based on *in situ* EXAFS data. The bulk phase structure of β-NiOOH is referenced to the structure type of EE (hydrogen atoms distributed on both sides) [5,6]. And as shown in **Supplementary Fig. 17a**, the simulated spectrum of the backscattering signal χ^3 (blue line) of the β-NiOOH (111) model in the inset matches well with the EXAFS data (grey line) of NiOx, evidencing the relative rationality of this NiOOH model. Similarly, the simulated spectrum of the backscattering signal χ^3 (red line) of the β-NiOOH (111)-SO_4 model also matches well with the EXAFS data (grey line) of Ni-SOx, which also confirms the relative rationality of this NiOOH-SO_4 model (**Supplementary Fig. 17b**).

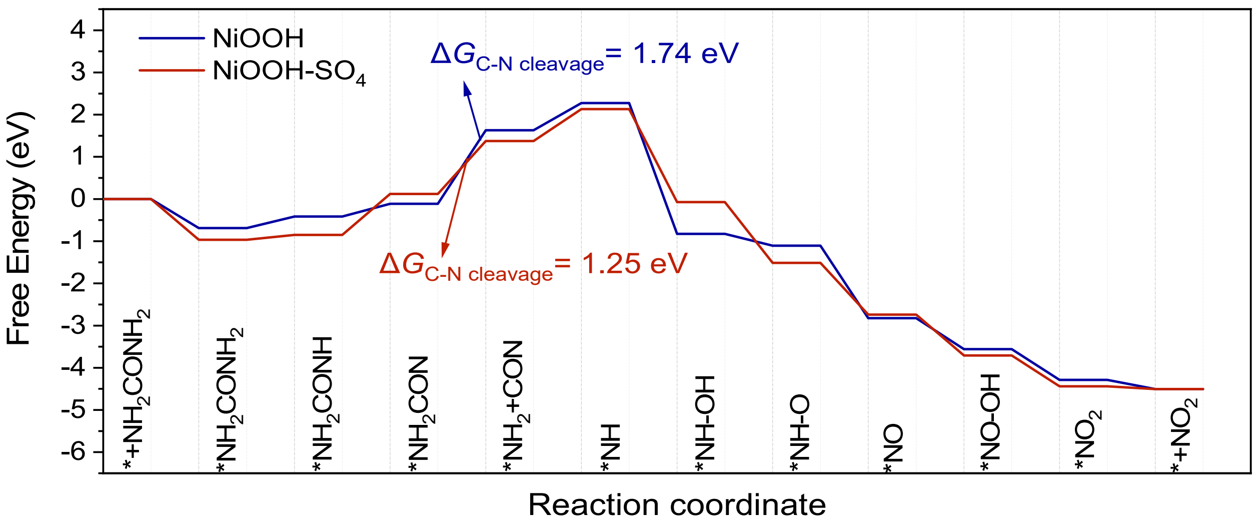

Supplementary Fig. 18 DFT calculations. Gibbs energy profiles of UOR in the alkaline (pH = 14) on NiOOH and $NiOOH-SO_4$ catalysts at U = 1.5 V vs RHE. The structures of the key intermediates in the reaction process are shown in Supplementary Table 3.

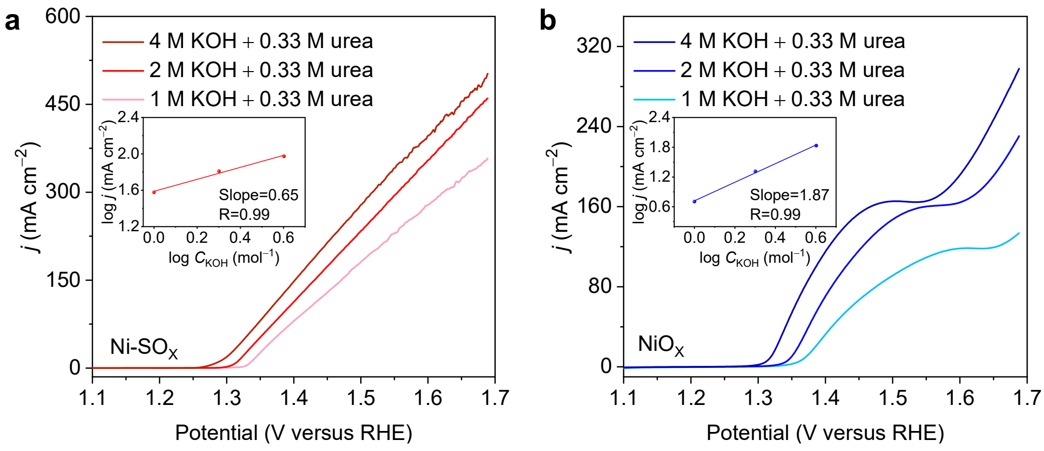

Supplementary Fig. 19 Polarization curves. LSV curves for (a) $Ni-SO_X$ and (b) NiO_X at different concentrations of KOH electrolyte containing 0.33 M urea. Inset: the dependence of UOR current density on KOH concentration at 1.36 V (vs. RHE).

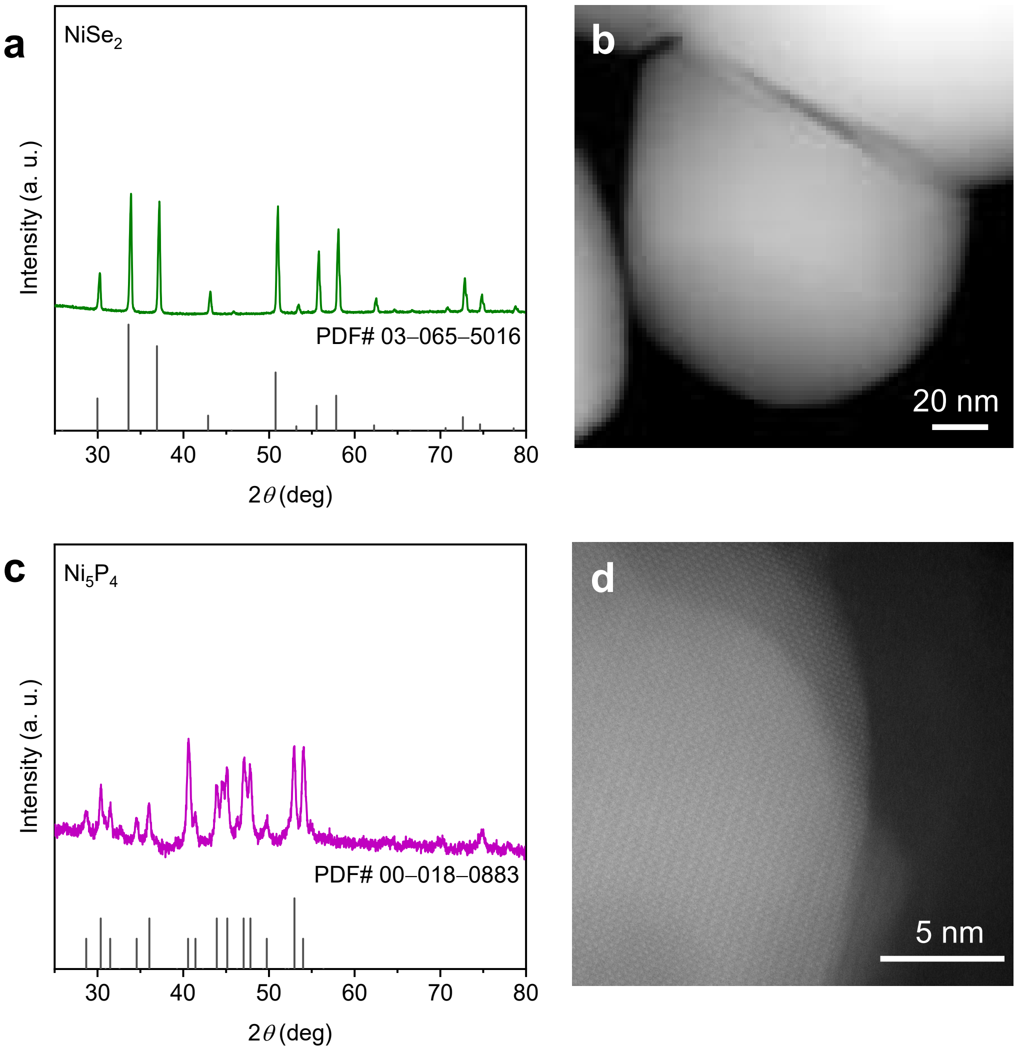

Supplementary Fig. 20 Structural characterization of $NiSe_2$ and Ni_5P_4. (a) XRD pattern and (b) HRTEM image, of $NiSe_2$. (c) XRD pattern and (d) HRTEM image, of Ni_5P_4.

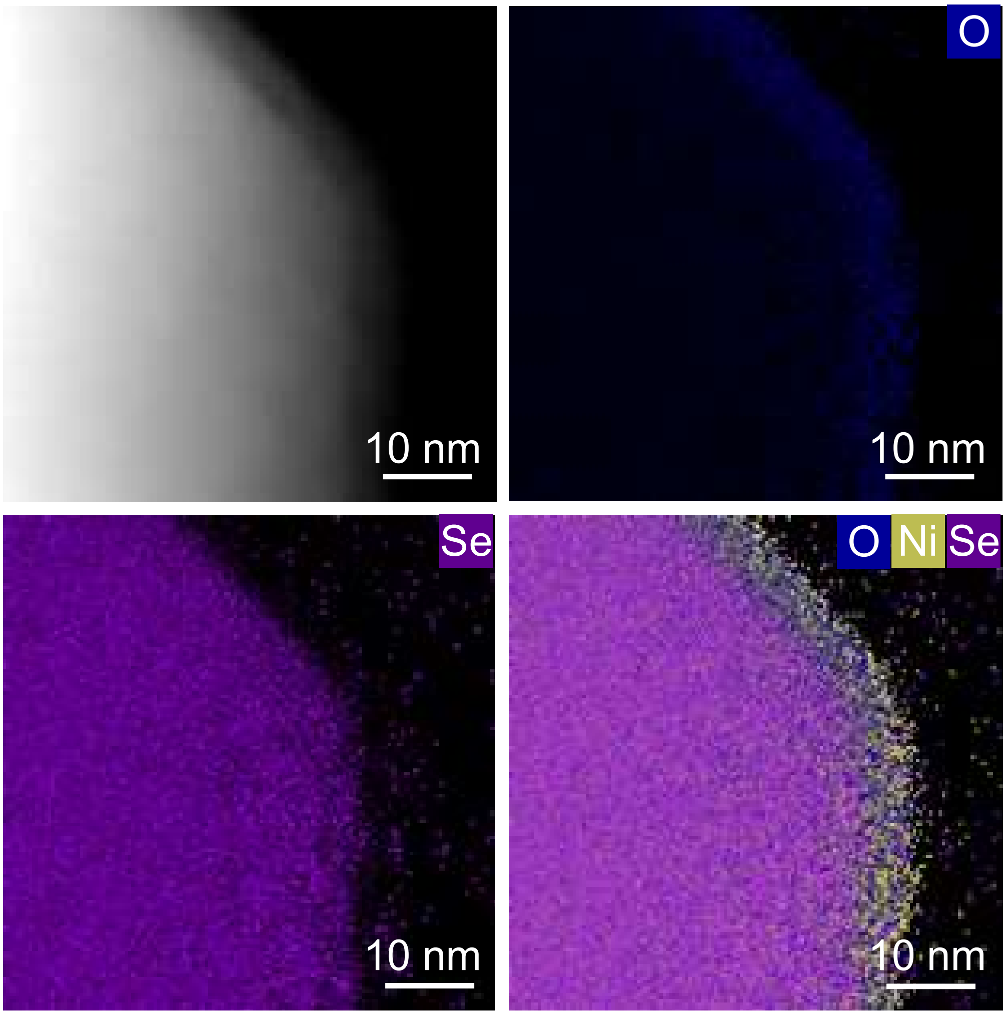

Supplementary Fig. 21 Structural characterization of Ni-SeOx. HADDF-STEM image and corresponding EDX elemental mapping for Ni-SeOx.

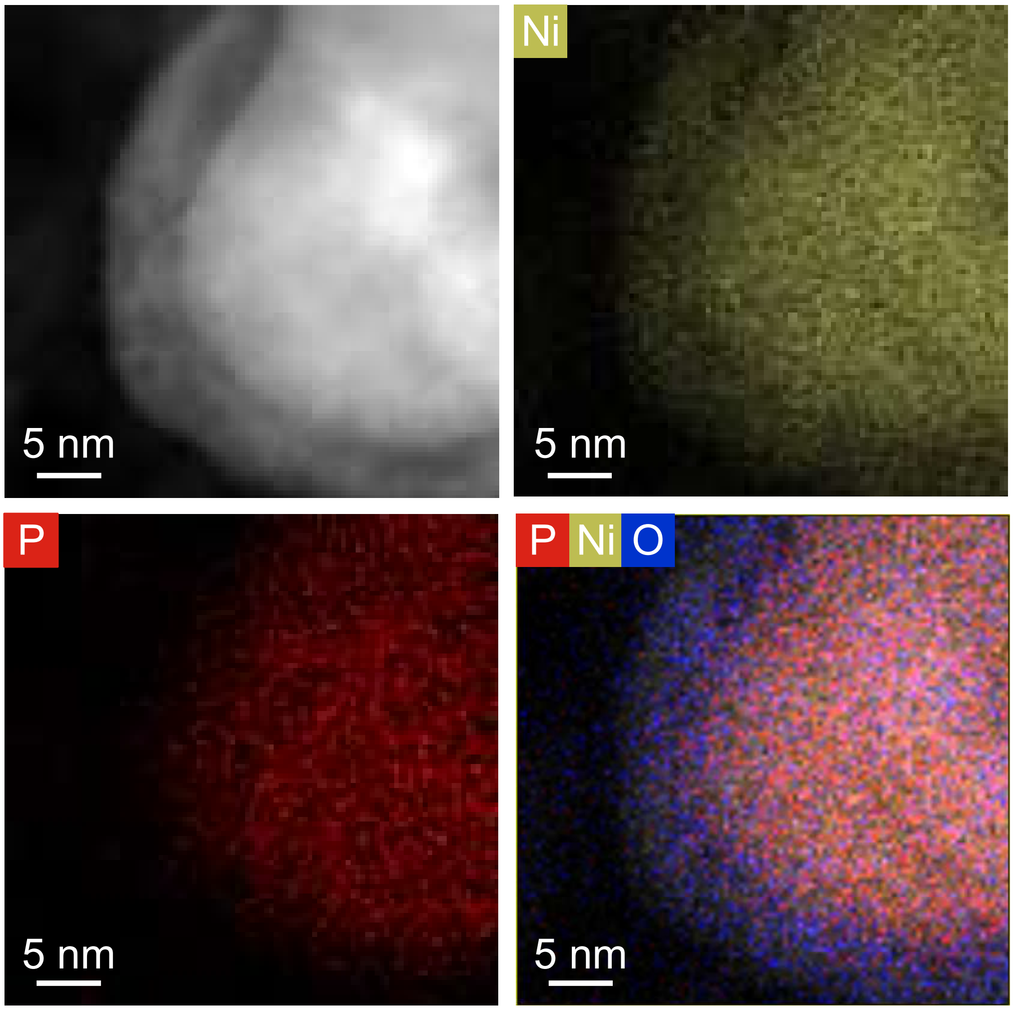

Supplementary Fig. 22 Structural characterization of Ni-PO_X. HADDF-STEM image and corresponding EDX elemental mapping for Ni-PO_X.

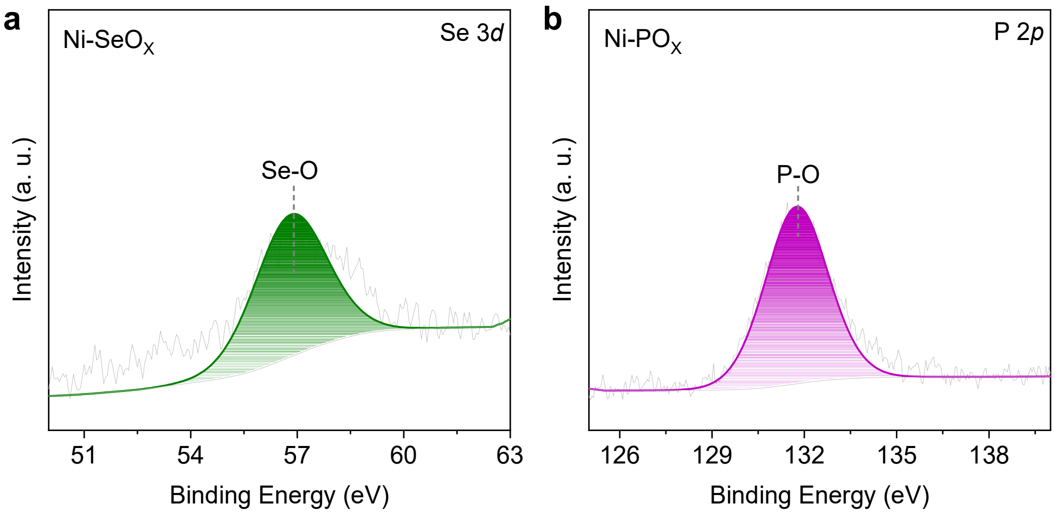

Supplementary Fig. 23 XPS characterizations. (a) Se *3d* XPS spectra for Ni-SeO_X. (b) P *2p* XPS spectra for Ni-PO_X.

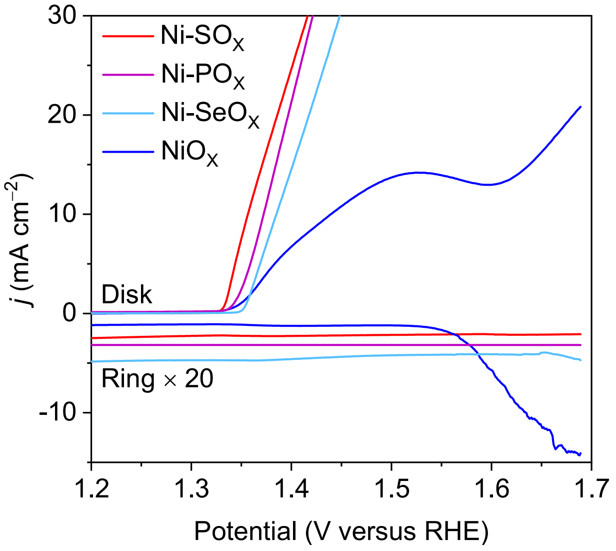

Supplementary Fig. 24 Electrocatalytic test. RRDE curves for NiO_X, Ni-SO_X, Ni-PO_X, and Ni-SeO_X in 1 M KOH with 0.33 M urea.

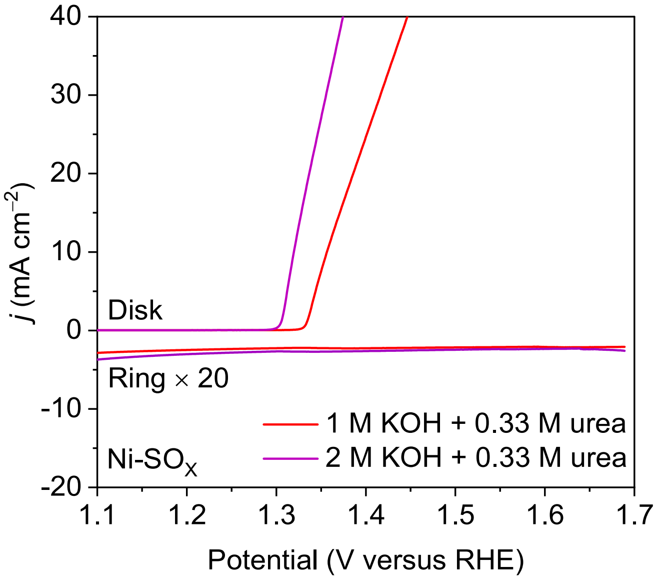

Supplementary Fig. 25 Electrocatalytic test. RRDE curves for Ni-SO_X obtained in different concentrations of KOH with 0.33 M urea.

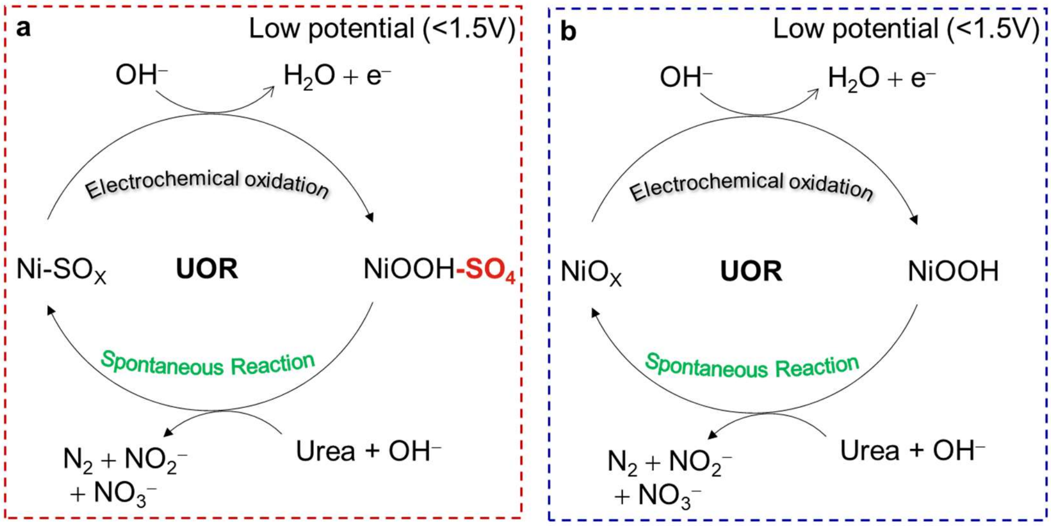

Supplementary Fig. 26 Mechanism analysis. Representation of UOR mechanism on (a) Ni-SO_X and (b) NiO_X accompanying adsorption of reactants and dynamic evolution of active site under low potential (< 1.50 V).

Supplementary Table 1. Electrocatalytic UOR performance for Ni-SO_X catalyst compared with recently reported powder catalysts.

Sample	Type of electrode	Electrolyte	Potential (V vs. RHE)	J (mA cm^{-2})	Ref.
Ni-SO_X	Carbon paper	1 M KOH + 0.33 M urea	1.65	323.4	This work
NiF_2/Ni_2P	Glass carbon	1 M KOH + 0.33 M urea	1.65	80.0	7
$Ni_{1.20}Co_{1.80}O_4$	Glass carbon	1 M KOH + 0.33 M urea	1.65	30.0	8
NiS/NiS_2	Carbon paper	1 M KOH + 0.33 M urea	1.50	120.5	9
$Ni_{1.6}Co_{0.4}P/C$	Glass carbon	1 M KOH + 0.33 M urea	1.60	140.0	10
Delithiated $LiNiO_2$	Glass carbon	1 M KOH + 0.50 M urea	1.50	5.0	11
Ni–Sn sulfide	Carbon paper	1 M KOH + 0.33M urea	1.65	150.0	12
Co, V co-doped NiS_2	Carbon paper	1 M KOH + 0.33 M urea	1.65	150.0	13
Ni@N-doped CNT	Glass carbon	1 M KOH + 0.50 M urea	1.60	80.0	14
$Ni_{0.85}Se$-on-rGO	Carbon paper	1 M KOH + 0.50 M urea	1.60	100.0	15
Ni-Co oxide	Glass carbon	1 M KOH + 0.33 M urea	1.50	60.0	16
Ni-MOF-0.5	Glass carbon	1 M KOH + 0.50 M urea	1.65	80.0	17
NiClO-D	Glass carbon	1 M KOH + 0.33 M urea	1.60	264.0	18
Ni/NiO@ N-doped C	Glass carbon	1 M KOH + 0.33 M urea	1.65	~80.0	19
Rh-NiO	Glass carbon	1 M KOH + 0.33 M urea	1.50	52.1	20
$Ni(OH)_2$ nanomeshes	Glass carbon	1 M KOH + 0.33 M urea	1.65	~60.0	21
NiS_2-MoS_2	Glass carbon	1 M KOH + 0.33 M urea	1.54	103.4	22
$NiSe_2$-NiO	Glass carbon	1 M KOH + 0.33 M urea	1.53	176.8	23
Pomegranate-like Ni/C	Glass carbon	1 M KOH + 0.50 M urea	1.65	~30.0	24

Supplementary Table 2. Electrocatalytic UOR selectivity for Ni-SO_X catalyst compared with recently reported catalysts.

Sample	Electrolyte	Potential	J (mA cm^{-2})	$FE_{N\ products}$ (%)	FE_{O2} (%)	$S_{N\ products}$ (%)	Ref.
Ni-SO_X	1 M KOH + 0.33 M urea	1.65 V vs. RHE	323.4	97.0	Negligible	99.3	This work
Ni nanowires	1 M KOH + 0.33 M Urea	1.55 V cell voltage	~33	75.7	19.5	79.5	25
$NiCo_2O_4$ nanosheets	5 M KOH + 0.33M Urea	0.50 V vs. Hg/HgO	40	89.0	11.0	89.0	26
NiCo-LDH-NO_3	1 M KOH + 0.33 M Urea	1.50V cell voltage	~6	85.6	~14.4	85.6	27
Ni foil	5 M KOH + 0.33 M Urea	1.55 V cell voltage	20	96.1	1.9	98.1	28
Activated Ni foam	1 M KOH + 0.33 M urea	0.60 V vs. Ag/AgCl	~40	50.9	Not quantified	N/A	29
NiCo bimetallic hydroxide	1 M KOH + 0.33 M Urea	1.50 V cell voltage	~18	78.0	Not quantified	N/A	30
$Ni(OH)_2$ nanocup arrays	1 M KOH + 0.33 M Urea	0.45 V vs. Hg/HgO	~150	82.2	17.8	82.2	31
RhNi	1 M KOH + 0.33 M Urea	1.55 V cell voltage	~23	93.0	Not quantified	N/A	32
NiCo bimetallic nanowire	1 M KOH + 0.33 M Urea	1.55 V cell voltage	~27	81.0	Not quantified	N/A	33
Bulk Ni film	1 M KOH + 0.33 M Urea	1.55 V cell voltage	~10	54.0	Not quantified	N/A	33

Supplementary Table 3. Summary of optimized configurations of UOR and OER intermediates on NiOOH(111) and NiOOH(111)-SO_4. The red, gray, light pink, blue, brown, and yellow spheres represent O, Ni, H, N, C, and S atoms, respectively.

Species	NiOOH(111)	NiOOH(111)-SO_4
*NH_2CONH_2	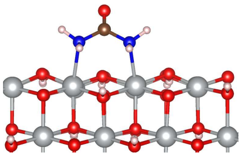	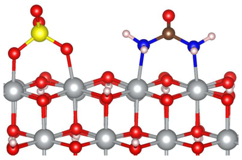
*NH_2CONH	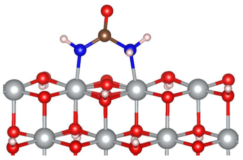	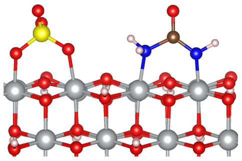
*NH_2CON	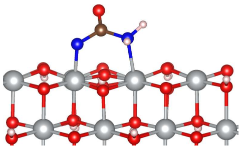	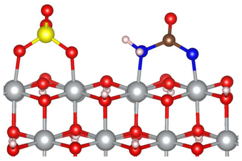
*NH_2	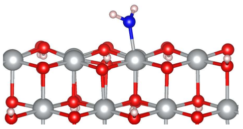	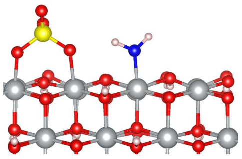
*NH	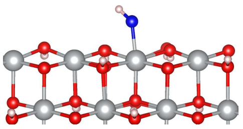	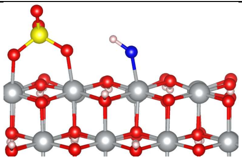
*NH-OH	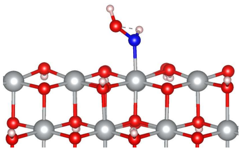	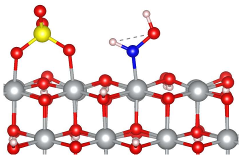
*NH-O	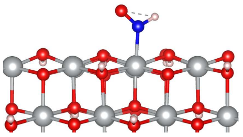	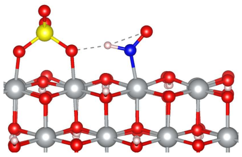
*NO	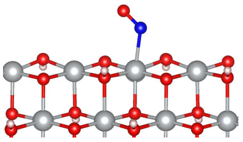	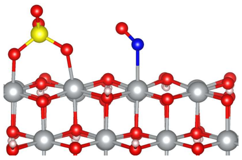
*NO-OH	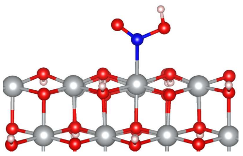	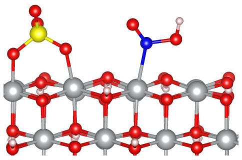

*NO_2	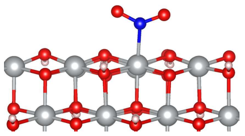	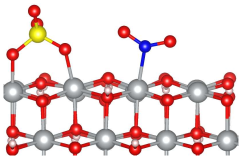

Supplementary note 1: Computation and models.

Spin-polarized DFT calculations were performed using the Vienna *ab initio* Simulation Package (VASP), a plane-wave pseudopotential code[34,35]. The Perdew-Burke-Ernzerhof (PBE) functional within the generalized gradient approximation (GGA) was used to describe electronic exchange and correlation[36]. All structures in the calculations were explored using PBE+U, with an effective U_{eff} = U-J term of 5.5 eV, for the Ni 3d state[37-39]. A planewave cut-off energy of 400 eV was set. During geometric optimization, all atoms were permitted to relax. The convergence criteria were 0.02 eV/Å and 10^{-5} eV for energy and force, respectively, for relaxing the structure. Van der Waals interaction was used for dispersion correction using DFT-D3 method[40,41]. A 1×1×1 Gamma-centered k-mesh was used in all systems. To analyze the effect of applied potential on adsorbates, the constant potential approach was used with the code reported by Zhao *et al.*[42, 43]. In this work, we use the implicit solvation model as implemented in VASPsol.

The (111) plane of an irreducible triclinic unit cell of *β*-NiOOH (i.e., the structure type of EE[5,6]) was selected as the model in **Supplementary Fig. 17**. Hydrogen atoms are distributed on both sides. For NiOOH-SO_4, the two O atoms of -SO_4 are bonded to two Ni atoms. The total atomic numbers of the NiOOH and NiOOH- SO_4 structures are 128 and 133, respectively. During geometric optimization, the top two layers of atoms were relaxed, and the bottom two layers are fixed. A 15 Å vacuum layer in the Z-axis was set to obviate interactions between structural periods. Atomic coordinates of *β*-NiOOH, NiOOH and NiOOH-SO_4 structures are provided in Supplementary note 2.

The UOR pathway:

$*+NH_2CONH_2 \rightarrow *NH_2CONH_2$

$*NH_2CONH_2+OH^- \rightarrow *NH_2CONH+H_2O+e^-$

$*NH_2CONH+OH^- \rightarrow *NH_2CON+H_2O+e^-$

$*NH_2CON \rightarrow *NH_2+CON$

$*NH_2+OH^- \rightarrow *NH+H_2O+e^-$

$*NH+OH^- \rightarrow *NH\text{-}OH+e^-$

$*NH\text{-}OH+OH^- \rightarrow *NH\text{-}O+H_2O+e$

$*NH\text{-}O+OH^- \rightarrow *NO+H_2O+e^-$

$*NO+OH^- \rightarrow *NO\text{-}OH+e^-$

$*NO\text{-}OH+OH^- \rightarrow *NO_2+H_2O+e^-$

$*NO_2 \rightarrow *+NO_2$

Taking the $*NH_2CONH_2+OH^- \rightarrow *NH_2CONH+H_2O+e^-$ reaction as an example, the free energy under the constant potential method is calculated as follows. In the RHE the following thermodynamic equilibrium exists, namely:[43]

$$G(H^+) + \mu_e(0_{RHE}) = G(H_2)/2 \quad (1)$$

$$\mu_e(U_{RHE}) = \mu_e(0_{RHE}) - |e|U_{RHE} \quad (2)$$

For $H_2O = H^+ + OH^-$ in equilibrium, $G(H_2O) = G(H^+) + G(OH^-) = G(H_2)/2 - \mu_e(0_{RHE}) + G(OH^-)$, therefore:

$$G(OH^-) = G(H_2O) - G(H_2)/2 + \mu_e(0_{RHE}) \quad (3)$$

Taking $^*NH_2CONH_2 + OH^- = {}^*NHCONH_2 + H_2O + e^-$ as an example, the Gibbs free energy at a certain potential was computed from:

$$\Delta G = G(^*NHCONH_2{}^{Q2}) - G(^*NH_2CONH_2{}^{*Q1}) + G(H_2O) + \mu_e(U_{RHE}) - G(OH^-) + (Q2 - Q1)\mu_e(U_{RHE}) \quad (4)$$

Substituting Eqs. 2 and 3 into Eq. 4, we have:

$$\Delta G = G(^*NHCONH_2{}^{Q2}) - G(^*NH_2CONH_2{}^{Q1}) + G(H_2)/2 + (Q2 - Q1)\mu_e(U_{RHE}) - |e|U_{RHE} \quad (5)$$

Supplementary note 2: Crystal Structure Data.

Here, we list the bulk phase structures of *β*-NiOOH as well as the structures of NiOOH and NiOOH-SO_4 used in the study.

Bulk phase structure of *β*-NiOOH

```
O4H2Ni2
  1.00000000000000
   2.8614417442712718  -0.0064338663252991   0.0090316163962445
   1.1926807220645053   4.5896242280292583  -0.0102082423284524
  -0.0942252659525459  -1.1586483946398969   4.8669118289616451
 O   H   Ni
   4   2   2
Direct
  0.9277658754549349  0.1977642900992730  0.7160416308680887
  0.3716252851154638  0.2394578404470072  0.2037742945043836
  0.0722416877826366  0.8022197273199643  0.2839544125960700
  0.6283347406208443  0.7606262920403221  0.7962362567468311
  0.7573320147296924  0.5341903601534934  0.7701397012467752
  0.2426990748374873  0.4657537492988096  0.2298554217740262
 -0.0000003463974430 -0.0000166187046727 -0.0000041533033532
  0.5000016678563837 -0.0000056406541935  0.5000024355671778
```

NiOOH

O64H32Ni32

1.00000000000000

9.7444000000000006 0.0000000000000000 0.0000000000000000

0.0000000000000007 11.5442000000000000 0.0000000000000000

0.0000000000000015 0.0000000000000015 24.2012000000000000

O H Ni

64 32 32

Direct

0.7975984508978455 0.4285204047823734 0.0582032591558707

0.8906078936725490 0.0573395579664000 0.1647452568052857

0.9918037687391081 0.1784558916900211 0.2581460697573108

0.0917248905163883 0.3042565974046966 0.3547501239761819

0.7974272946942765 0.1851152436268148 0.0581166065399093

0.8704919269784067 0.3058857426054145 0.1632791329274291

0.9747001197896416 0.4364876662381493 0.2562264286557365

0.0806194088398588 0.0562810003582606 0.3577738536937518

0.0340355540446985 0.1872879241876678 0.0982597315197007

0.1233530951896468 0.3081790217175404 0.1942440540849531

0.2264270577055813 0.4327733626302667 0.2873404547623972

0.3232402252272487 0.0536598776162447 0.3884992731590498

0.1424683095770840 0.0550059625619415 0.1930350496176094

0.0336485955512874 0.4261834203951593 0.0981944468452829

0.2434567968264102 0.1760482593890211 0.2898657325087612

0.3265747621600226 0.3056755741326214 0.3935637629983700

0.2942611936633113 0.4322514754320103 0.0597710534073671

0.3902794237296912 0.0572642331667448 0.1647291776378722

0.4920694780682589 0.1788643572695537 0.2581745786967869

0.5924638390107743 0.3041477536973318 0.3548329132975866

0.2945396658818015 0.1818979495234413 0.0601308307606172

0.3706729997488331 0.3076715180270850 0.1628292359026109

0.4754958544374947 0.4370294111852372 0.2573119352498613

0.5817434593620534 0.0562992631496613 0.3582846763898903

0.5297924662485568	0.1843874051363237	0.0966169735803420
0.6224975789671492	0.3086738943033038	0.1935288687609603
0.7268005817393695	0.4325423418521311	0.2870987278701150
0.8249022115230512	0.0536637850299151	0.3880258394907249
0.6420250827340811	0.0543158572870489	0.1924301610171702
0.5292791330912001	0.4299853283914294	0.0953611624523344
0.7429484867154235	0.1753928657136098	0.2895219246221342
0.8275622759888399	0.3055621400679967	0.3932190357775665
0.7975984429138110	0.9285210285347337	0.0582033452847353
0.8906080507746886	0.5573394612744781	0.1647448609954943
0.9918037658118344	0.6784558659193043	0.2581459746843853
0.0917249031728448	0.8042565726803054	0.3547501356685935
0.7974272319801953	0.6851158305549417	0.0581165192296072
0.8704918926837091	0.8058859981355576	0.1632793730463271
0.9746998585627439	0.9364877306290716	0.2562271510354646
0.0806194689350006	0.5562809380721340	0.3577738876713544
0.0340357406092699	0.6872883883772714	0.0982597103382153
0.1233528886178348	0.8081793847798335	0.1942443382313443
0.2264270991709304	0.9327733302649341	0.2873405256857970
0.3232402545027626	0.5536598491904722	0.3884992122895572
0.1424683282982785	0.5550057313425230	0.1930347724836234
0.0336481096503149	0.9261840512319446	0.0981949501635595
0.2434567562216861	0.6760481286300062	0.2898656930644704
0.3265747791547420	0.8056754634392610	0.3935637767180371
0.2942612351351786	0.9322516130689870	0.0597709635039865
0.3902794325464126	0.5572642580257949	0.1647291973215389
0.4920694998413508	0.6788643646528759	0.2581745451546573
0.5924638023885258	0.8041477403455815	0.3548329181943911
0.2945395136535046	0.6818979783969494	0.0601309060070076
0.3706728798987584	0.8076717038639876	0.1628292425401316
0.4754958657517621	0.9370294907347232	0.2573120346580258
0.5817433771608933	0.5562992168667353	0.3582846521748554
0.5297922610348619	0.6843875435507782	0.0966170150987570

0.6224975576149967	0.8086739771102031	0.1935289032127648
0.7268005744219146	0.9325423463867351	0.2870987682183605
0.8249021770745876	0.5536637248740308	0.3880257727721276
0.6420252533788670	0.5543159954045239	0.1924301716132348
0.5292792075151279	0.9299854017783152	0.0953610784241508
0.7429486595674329	0.6753928396652578	0.2895219972213939
0.8275622002802716	0.8055620836592551	0.3932190326026648
0.2443863727275251	0.0548209455776375	0.1815721457010125
0.1978783360624672	0.4301955220721668	0.0758034900506416
0.3433553162255962	0.1732887428886387	0.2765403827280743
0.7005510909422406	0.1866854809445668	0.0724550831667936
0.7701450801676721	0.3065686266908994	0.1759047680129074
0.8724708195253378	0.4385212146011597	0.2680582756429917
0.9781128607875265	0.0557965733753122	0.3716132210979898
0.4233351472290184	0.3055730432870310	0.3791241540604985
0.7438533752919112	0.0538390847864842	0.1807377784775371
0.7002813605311194	0.4271024111992542	0.0720912151595643
0.8427329297751253	0.1721385798548587	0.2760389091608148
0.1977443546997896	0.1837071981706904	0.0758196465964033
0.2708267074335025	0.3082697676871057	0.1761757244572050
0.3738786245252419	0.4394985148098936	0.2694271762640820
0.4803784594112309	0.0558864317947974	0.3722712128521876
0.9244673802850154	0.3054375906862232	0.3788021093942441
0.2443863929150986	0.5548209986489027	0.1815720920344250
0.1978783320566246	0.9301958035719899	0.0758034442356224
0.3433553009505655	0.6732887213693517	0.2765403123533853
0.7005510658918263	0.6866859900950403	0.0724550063661458
0.7701452332285921	0.8065686422760878	0.1759047671113613
0.8724703806597877	0.9385211525828414	0.2680584606540522
0.9781128712185887	0.5557965538467344	0.3716131658478149
0.4233351762474803	0.8055729228573213	0.3791241785941866
0.7438534792753123	0.5538390257873790	0.1807377738997333
0.7002812807792885	0.9271030151757333	0.0720913056214998

0.8427329441300399	0.6721386847211999	0.2760388836748202
0.1977441938023184	0.6837074593322381	0.0758197668066064
0.2708267608228567	0.8082701039691111	0.1761756966991512
0.3738786491508809	0.9394985442354173	0.2694272850509453
0.4803783653611793	0.5558862347919401	0.3722711388124262
0.9244673003998943	0.8054376000891926	0.3788021217044651
0.9082530621366939	0.3066353886998490	0.0827807406364281
0.0053226379389034	0.4321125220177424	0.1774868285211288
0.1099722090341578	0.0559320891368386	0.2734374946621642
0.2140411790473082	0.1793504839275544	0.3701142494377499
0.9089136197355147	0.0569246582518776	0.0825988642865500
0.0082950601094851	0.1818794859194563	0.1778591664693506
0.1086337599767802	0.3058673097568720	0.2740945781619951
0.2129145706731868	0.4301588053992621	0.3691744372744249
0.4053610429057167	0.3071820845590413	0.0825319213986206
0.5054102204717569	0.4327241713555217	0.1772498140471485
0.6100158726906719	0.0558770497867817	0.2732243608105052
0.7149759141727718	0.1794019022165400	0.3700077761901308
0.4069383874915153	0.0570151915398022	0.0826528884372807
0.5077149622814833	0.1819914807849769	0.1774915128829892
0.6086087312171931	0.3060592762660270	0.2739913227713062
0.7139397631682152	0.4300507478160639	0.3690173452254437
0.9082531865698563	0.8066358576466014	0.0827808608438099
0.0053216181683047	0.9321124887929468	0.1774880083197093
0.1099720158917250	0.5559318855117658	0.2734373108977396
0.2140412251873658	0.6793504299091454	0.3701142141220588
0.9089134462871749	0.5569248978096348	0.0825986300789945
0.0082951157031523	0.6818795044205143	0.1778590695889478
0.1086339543874704	0.8058671051603785	0.2740946893519904
0.2129145913015081	0.9301587925077867	0.3691744705852626
0.4053611317394100	0.8071821116159125	0.0825319564348187
0.5054101930752709	0.9327242385643433	0.1772498207444749
0.6100159671300851	0.5558770814958726	0.2732243587132024

0.7149758230522106 0.6794018991819385 0.3700077797987116
0.4069383073414599 0.5570152083718050 0.0826528882737374
0.5077149966614884 0.6819914960969687 0.1774914877509730
0.6086087543990217 0.8060592830324593 0.2739913277647996
0.7139397878974608 0.9300507726935991 0.3690173759978196

NiOOH-SO4

O68H32Ni32S1
1.00000000000000
9.7444000000000006 0.0000000000000000 0.0000000000000000
0.0000000000000007 11.5442000000000000 0.0000000000000000
0.0000000000000015 0.0000000000000015 24.2012000000000000
O H Ni S
68 32 32 1
Direct
0.8011703285664263 0.4266993051855792 0.0605393757958447
0.8930555048169746 0.0517533058736151 0.1653436402103831
0.9905841148412453 0.1735056261010268 0.2587293902839436
0.0862055535722763 0.2988174685763734 0.3560027667984841
0.8017875552186233 0.1825836727602010 0.0587027625086256
0.8715490891612198 0.3009078956044254 0.1641324754286731
0.9742732314705200 0.4287532426747037 0.2569917305278718
0.0748656273554376 0.0523696895309979 0.3580944555420248
0.0378856015004576 0.1844638634277948 0.0994408134345874
0.1242792256883839 0.3026200478265896 0.1952490024329523
0.2266077626963208 0.4242880457121277 0.2884510931327864
0.3199834537727365 0.0509071892273258 0.3880737858692747
0.1454962100486857 0.0496021620856115 0.1946004653761147
0.0361942436018471 0.4244824536629063 0.1005180643787804
0.2415615913356876 0.1725435445523913 0.2912048385297168
0.3226003067717379 0.2991189899157372 0.3943123073254464
0.2992374513771543 0.4281446019162032 0.0617845574837723
0.3922204767774037 0.0510648605093538 0.1652081569259465

0.4906607707862671	0.1725989029832244	0.2592014553228514
0.5892911408617232	0.2993782403376320	0.3547301237897679
0.2987482514085863	0.1776881629911118	0.0609199559632955
0.3707988772360398	0.3000951942390350	0.1641097939669322
0.4745034632322577	0.4283899230754700	0.2577220187901288
0.5824309068757163	0.0507348766341486	0.3594701392090240
0.5327640768339015	0.1797046027870449	0.0979503950492223
0.6230540326287684	0.3019107101241701	0.1948755687312570
0.7294856853138245	0.4232774465270174	0.2865089869613362
0.8248472817140897	0.0497185388062530	0.3891654912919345
0.6430575964796837	0.0480371234881915	0.1930795408145918
0.5329645573358720	0.4257607067191183	0.0996768728442124
0.7419001065986084	0.1700752076314246	0.2904121883458292
0.8227684003427747	0.3015768174110897	0.3944458344911500
0.8031448595314571	0.9211301503569457	0.0589772008923441
0.8924827314747330	0.5532288898164853	0.1676389196487850
0.9971213229431208	0.6738038312596707	0.2602198234242648
0.0853540175811939	0.8056103477173582	0.3525015567445945
0.8012402341582275	0.6778892565505006	0.0609801167787204
0.8752624146941693	0.8009780868595083	0.1653208904712385
0.9774870545478048	0.9323485925813888	0.2567099969453377
0.0786613682363292	0.5433177300380183	0.3570231675319597
0.0371977646447851	0.6795205313680742	0.1007341476443308
0.1293675778019318	0.8031245713246977	0.1964815118206253
0.2284130129578995	0.9319030084622130	0.2928610118797470
0.3216394013790384	0.5395852901085826	0.3930482718494701
0.1464323765085732	0.5499217903988026	0.1975696310520635
0.0387597305827072	0.9193733859528865	0.1001400633750373
0.2472219308398448	0.6710781849174294	0.2961359017680227
0.3346177124711950	0.8141613826985590	0.3852511310385296
0.2986444264397969	0.9262979431660979	0.0601759831343011
0.3919488791772801	0.5530132705983627	0.1676633063730482
0.4958456137359131	0.6758396626872467	0.2606458796945002

0.5947008564452873	0.7998682393649180	0.3540754060214284
0.2992478464565416	0.6760763680557440	0.0621413335315476
0.3752413846382722	0.8052727426765737	0.1641623225210545
0.4790097350022596	0.9313896415739932	0.2597328286696740
0.5909395133961789	0.5511591218580116	0.3535537328763623
0.5338365618465162	0.6784476781354400	0.0997443295950284
0.6265793608551302	0.8041734092163846	0.1949522608913596
0.7309942509175589	0.9285464968839079	0.2881501717736938
0.8229069512657349	0.5475123617657462	0.3888169610718374
0.6453848416386799	0.5495547105824540	0.1978320605995373
0.5333916733574697	0.9248254161172184	0.0947688053734616
0.7503251352413657	0.6774596405842034	0.2890986729814600
0.8251471693580921	0.8001277186963633	0.3946089359172011
0.1456158803106115	0.6992291677269145	0.4499278677075500
0.0833682041323941	0.8103916574660689	0.5292580227697055
0.3227370184939334	0.8095557027121364	0.5001126718708898
0.1434902294916092	0.9140391765269181	0.4476247691323740
0.2472128868223588	0.0491785331171657	0.1826326006670771
0.2027146986035000	0.4269583883327577	0.0772663884469562
0.3416608032159847	0.1690275225702507	0.2777221985975916
0.7052385792128353	0.1844051242214800	0.0730833358805413
0.7713080437579222	0.3008611630697478	0.1766980458789379
0.8704622717272990	0.4279633257545553	0.2683390436729238
0.9709761717126705	0.0506248843238760	0.3732709408395860
0.4190744213424153	0.3000496009443275	0.3793886004628594
0.7446508038316754	0.0471048821317034	0.1809924882350825
0.7047337320207953	0.4253240802822633	0.0752958766174747
0.8415177518053819	0.1674815262207248	0.2767047115119689
0.2021275581318893	0.1802078192718256	0.0767788291418669
0.2705299111995732	0.3004399644152485	0.1769716541772394
0.3723480622656333	0.4279587902174034	0.2691661977023516
0.4822955462133920	0.0503272420375754	0.3734561505115133
0.9203058920886052	0.3010239452699953	0.3808186214238120

0.2491244369399477	0.5504313538093115	0.1860716245469825
0.2029298117875147	0.9237801493235553	0.0768383618273229
0.3473145233861623	0.6708291268546135	0.2830121503698464
0.7046392629145907	0.6791811815465437	0.0757377495844495
0.7746519718186433	0.8023703648877408	0.1777398028996757
0.8746192100564616	0.9336138806032686	0.2687590962893033
0.9775369612224628	0.5410857813588920	0.3704649702514517
0.4291377496854203	0.8181108054154398	0.3684223702224262
0.7483823550140382	0.5502366498248860	0.1866997459456508
0.7048125695704389	0.9194407117328695	0.0715352659406651
0.8511209842817723	0.6757999381336540	0.2767204836528726
0.2026305841831893	0.6772346997084645	0.0776332815856752
0.2752645653086792	0.8065942679607947	0.1778495971754328
0.3802775512118153	0.9355912010283179	0.2739618844936348
0.4170156754903829	0.5390128348296018	0.3777507787267220
0.9225771989986665	0.8019424229305242	0.3808186341501817
0.9119710915105540	0.3031187036628689	0.0841713841675279
0.0066301834288325	0.4270423248074531	0.1795069905061381
0.1095772907430006	0.0524578073327978	0.2750515893101796
0.2088947214916287	0.1735922208071022	0.3714711062202800
0.9129445009936201	0.0521071073112335	0.0833175961530012
0.0098144958870389	0.1768046804796495	0.1787131461079534
0.1068165170186830	0.3005252535102820	0.2751528610468026
0.2081928364873370	0.4202245615858241	0.3708689922164535
0.4090893493405962	0.3021995236908949	0.0844086220439989
0.5054945958729390	0.4266707436634443	0.1795887548292591
0.6111837550250458	0.0505516586415437	0.2745111561087519
0.7127762152427125	0.1753161422644854	0.3712772760112814
0.4108461767480267	0.0517022680237566	0.0831056490333268
0.5083560823786891	0.1760806306999200	0.1787312515258102
0.6077596147910237	0.2999109440007290	0.2748345112066032
0.7129771063689581	0.4265042121578683	0.3688991498148965
0.9126652357524050	0.8005096605203810	0.0847590206636760

0.0092404642105512	0.9270309703316478	0.1786372692097602
0.1121006534843819	0.5493409646753304	0.2761807598619355
0.2113020614952996	0.6791075130903168	0.3748228791488932
0.9123786967589689	0.5522706222063488	0.0854820279010224
0.0126752669011093	0.6767566532517032	0.1805471333867274
0.1123398743771977	0.8025812286999251	0.2758621273755455
0.2044366119720306	0.9277062897533004	0.3719927908068848
0.4100744284623144	0.8021473260101526	0.0837109299058353
0.5087886927708516	0.9275168000792304	0.1781808363619850
0.6157160605504552	0.5507853833094596	0.2752098290057309
0.7165683598584711	0.6730134007023627	0.3696423396260836
0.4108547228747436	0.5522854483170668	0.0856104496109776
0.5104762045639277	0.6775648095970704	0.1803201990681998
0.6139799820768033	0.8018167812001883	0.2749172802055880
0.7146218206426794	0.9246135948522907	0.3703169413480520
0.1795622653220693	0.8078240329303082	0.4830479558529802

References

1. Lyu, S. et al. Exceptional catalytic activity of oxygen evolution reaction via two-dimensional graphene multilayer confined metal-organic frameworks. *Nat. Commun.* **13**, 6171 (2022).

2. You, H. et al. Monolayer NiIr-layered double hydroxide as a long-lived efficient oxygen evolution catalyst for seawater splitting. *J. Am. Chem. Soc.* **144**, 9254-9263 (2022).

3. Li, S. et al. Coordination environment tuning of nickel sites by oxyanions to optimize methanol electro-oxidation activity. *Nat. Commun.* **13**, 2916 (2022).

4. Zhao, T. et al. In situ reconstruction of V-doped Ni_2P pre-catalysts with tunable electronic structures for water oxidation. *Adv. Funct. Mater.* **31**, 2100614 (2021).

5. He, Z.-D., Tesch, R., Eslamibidgoli, M. J., Eikerling, M. H. & Kowalski, P. M. Low-spin state of Fe in Fe-doped NiOOH electrocatalysts. *Nat. Commun.* **14**, 3498 (2023).

6. Martirez, J. M. P. & Carter, E. A. Effects of the aqueous environment on the stability and chemistry of β-NiOOH surfaces. *Chem. Mater.* **30**, 5205-5219 (2018).

7. Liu, H., Liu, Z. & Feng, L. Bonding state synergy of the NiF_2/Ni_2P hybrid with the co-existence of covalent and ionic bonds and the application of this hybrid as a robust catalyst for the energy-relevant electrooxidation of water and urea. *Nanoscale* **11**, 16017-16025 (2019).

8. Li, Y. et al. Spin state tuning of the octahedral sites in Ni–Co-based spinel toward highly efficient urea oxidation reaction. *J. Phys. Chem. C* **125**, 9190-9199 (2021).

9. Liu, H., Liu, Z., Wang, F. & Feng, L. Efficient catalysis of N doped NiS/NiS_2 heterogeneous structure. *Chem. Eng. J.* **397**, 125507 (2020).

10. Rezaee, S. & Shahrokhian, S. 3D ternary $Ni_xCo_{2-x}P/C$ nanoflower/nanourchin arrays grown on HCNs: a highly efficient bi-functional electrocatalyst for boosting hydrogen production via the urea electro-oxidation reaction. *Nanoscale* **12**, 16123-16135 (2020).

11. Han, W.-K. et al. Activating lattice oxygen in layered lithium oxides through cation vacancies for enhanced urea electrolysis. *Angew. Chem. Int. Ed.* **61**, e202206050 (2022).

12. Ji, Z. et al. Accurate synergy effect of Ni–Sn dual active sites enhances electrocatalytic oxidation of urea for hydrogen evolution in alkaline medium. *J. Mater. Chem. A* **8**, 14680-14689 (2020).

13. Ji, Z. et al. Pathway manipulation via Ni, Co, and V ternary synergism to realize high efficiency for urea electrocatalytic oxidation. *ACS Catal.* **12**, 569-579 (2022).

14. Zhang, Q. et al. Nitrogen dopants in nickel nanoparticles embedded carbon nanotubes promote overall urea oxidation. *Appl. Catal. B: Environ.* **280**, 119436 (2021).

15. Zhao, L., Chang, Y., Jia, M., Jia, J. & Wen, Z. Monodisperse $Ni_{0.85}Se$ nanocrystals on rGO for high-performance urea electrooxidation. *J. Alloys Compd.* **852**, 156751 (2021).

16. Wang, S., Yang, X., Liu, Z., Yang, D. & Feng, L. Efficient nanointerface hybridization in a nickel/cobalt oxide nanorod bundle structure for urea electrolysis. *Nanoscale* **12**, 10827-10833 (2020).

17. Zheng, S., Zheng, Y., Xue, H. & Pang, H. Ultrathin nickel terephthalate nanosheet three-dimensional aggregates with disordered layers for highly efficient overall urea electrolysis. *Chem. Eng. J.* **395**, 125166 (2020).

18. Zhang, L. et al. A lattice-oxygen-involved reaction pathway to boost urea oxidation. *Angew. Chem. Int. Ed.* **131**, 16976-16981 (2019).

19. Ji, X., Zhang, Y., Ma, Z. & Qiu, Y. Oxygen vacancy-rich Ni/NiO@NC nanosheets with schottky heterointerface for efficient urea oxidation reaction. *ChemSusChem* **13**, 5004-5014 (2020).

20. Ma, G. et al. Ultrafine Rh nanocrystals decorated ultrathin NiO nanosheets for urea electro-oxidation. *Appl. Catal. B: Environ.* **265**, 118567 (2020).

21. Ding, Y. et al. Atomically thick $Ni(OH)_2$ nanomeshes for urea electrooxidation. *Nanoscale* **11**, 1058-1064 (2019).

22. Wang, S. et al. High valence state of Ni and Mo synergism in NiS_2-MoS_2 hetero-nanorods catalyst with layered surface structure for urea electrocatalysis. *J. Energy Chem.* **66**, 483-492 (2022).

23. Liu, Z., Zhang, C., Liu, H. & Feng, L. Efficient synergism of $NiSe_2$ nanoparticle/NiO nanosheet for energy-relevant water and urea electrocatalysis. *Appl. Catal. B: Environ.* **276**, 119165 (2020).

24. Wang, L. et al. Multivariate MOF-templated pomegranate-like Ni/C as efficient bifunctional electrocatalyst for hydrogen evolution and urea oxidation. *ACS Appl. Mater. Interfaces.* **10**, 4750-4756 (2018).

25. Yan, W., Wang, D., Diaz, L. A. & Botte, G. G. Nickel nanowires as effective catalysts for urea electro-oxidation. *Electrochim. Acta.* **134**, 266-271 (2014).

26. Wang, D., Vijapur, S. H., Wang, Y. & Botte, G. G. $NiCo_2O_4$ nanosheets grown on current collectors as binder-free electrodes for hydrogen production via urea electrolysis. *Int J Hydrogen Energy* **42**, 3987-3993 (2017).

27. Zeng, M. et al. Interlayer Effect in NiCo Layered double hydroxide for promoted electrocatalytic urea oxidation. *ACS Sustain. Chem. Eng.* **7**, 4777-4783 (2019).

28. Boggs, B. K., King, R. L. & Botte, G. G. Urea electrolysis: direct hydrogen production from urine. *Chem. Commun.*, 4859-4861 (2009).

29. Li, J. et al. Deciphering and suppressing over-oxidized nitrogen in Nickel-catalyzed urea electrolysis. *Angew. Chem. Int. Ed.* **133**, 26860-26866 (2021).

30. Yan, W., Wang, D. & Botte, G. G. Nickel and cobalt bimetallic hydroxide catalysts for urea electro-oxidation. *Electrochim. Acta.* **61**, 25-30 (2012).

31. Wu, M.-S., Ji, R.-Y. & Zheng, Y.-R. Nickel hydroxide electrode with a monolayer of nanocup arrays as an effective electrocatalyst for enhanced electrolysis of urea. *Electrochim. Acta.* **144**, 194-199 (2014).

32. King, R. L. & Botte, G. G. Investigation of multi-metal catalysts for stable hydrogen production via urea electrolysis. *J. Power Sources* **196**, 9579-9584 (2011).

33. Yan, W., Wang, D. & Botte, G. G. Template-assisted synthesis of Ni−Co bimetallic nanowires for urea electrocatalytic oxidation. *J. Appl. Electrochem.* **45**, 1217-1222 (2015).

34. Kresse, G. & Hafner, J. *Ab initio* molecular dynamics for liquid metals. *Phys. Rev. B* **47**, 558-561 (1993).

35. Kresse, G. & Furthmüller, J. Efficiency of *ab-initio* total energy calculations for metals and semiconductors using a plane-wave basis set. *Comput. Mater. Sci.* **6**, 15-50 (1996).

36. Perdew, J. P., Burke, K. & Ernzerhof, M. Generalized gradient approximation made simple. *Phys. Rev. Lett.* **77**, 3865-3868 (1996).

37. Li, Y.-F., Li, J.-L. & Liu, Z.-P. Structure and catalysis of NiOOH: recent advances on atomic simulation. *J. Phys. Chem. C.* **125**, 27033-27045 (2021).

38. Liu, J. et al. Structural and electronic engineering of Ir-doped Ni-(Oxy)hydroxide nanosheets for enhanced oxygen evolution activity. *ACS Catal.* **11**, 5386-5395 (2021).

39. Hu, Q. et al. Structure and oxygen evolution activity of β-NiOOH: Where are the protons? *ACS Catal.* **12**, 295-304 (2022).

40. Grimme, S., Antony, J., Ehrlich, S. & Krieg, H. A consistent and accurate *ab initio* parametrization of density functional dispersion correction (DFT-D) for the 94 elements H-Pu. *J. Chem. Phys.* **132**, 154104 (2010).

41. Grimme, S., Ehrlich, S. & Goerigk, L. Effect of the damping function in dispersion corrected density functional theory. *J. Comput. Chem.* **32**, 1456-1465 (2011).

42. Zhao, X. & Liu, Y. Origin of selective production of hydrogen peroxide by electrochemical oxygen reduction. *J. Am. Chem. Soc.* **143**, 9423-9428 (2021).

43. Zhao, X., Levell, Z. H., Yu, S. & Liu, Y. Atomistic understanding of two-dimensional electrocatalysts from first principles. *Chem. Rev.* **122**, 10675-10709 (2022).
